# Homology blocks of *Plasmodium falciparum var* genes and clinically distinct forms of severe malaria in a local population

**DOI:** 10.1186/1471-2180-13-244

**Published:** 2013-11-06

**Authors:** Mary M Rorick, Thomas S Rask, Edward B Baskerville, Karen P Day, Mercedes Pascual

**Affiliations:** 1 Department of Ecology and Evolutionary Biology, University of Michigan, 2019 Kraus Nat. Sci. Bldg., 830 North University Ave, Ann Arbor 48109-1048, Michigan, USA; 2 Howard Hughes Medical Institute, Ann Arbor, Michigan, USA; 3 Department of Microbiology, Division of Medical Parasitology, New York University School of Medicine, New York City, New York, USA; 4 Center for Computational Medicine and Bioinformatics, University of Michigan, Ann Arbor, Michigan, USA

**Keywords:** *Plasmodium falciparum*, Malaria, PfEMP1, *Var*, Severe disease, Homology block, Rosetting, Impaired consciousness, Recombination, Balancing selection

## Abstract

**Background:**

The primary target of the human immune response to the malaria parasite *Plasmodium falciparum*, *P. falciparum* erythrocyte membrane protein 1 (PfEMP1), is encoded by the members of the hyper-diverse *var* gene family. The parasite exhibits antigenic variation via mutually exclusive expression (switching) of the ~60 *var* genes within its genome. It is thought that different variants exhibit different host endothelial binding preferences that in turn result in different manifestations of disease.

**Results:**

*Var* sequences comprise ancient sequence fragments, termed homology blocks (HBs), that recombine at exceedingly high rates. We use HBs to define distinct *var* types within a local population. We then reanalyze a dataset that contains clinical and *var* expression data to investigate whether the HBs allow for a description of sequence diversity corresponding to biological function, such that it improves our ability to predict disease phenotype from parasite genetics. We find that even a generic set of HBs, which are defined for a small number of non-local parasites: capture the majority of local sequence diversity; improve our ability to predict disease severity from parasite genetics; and reveal a previously hypothesized yet previously unobserved parasite genetic basis for two forms of severe disease. We find that the expression rates of some HBs correlate more strongly with severe disease phenotypes than the expression rates of classic *var* DBLα tag types, and principal components of HB expression rate profiles further improve genotype-phenotype models. More specifically, within the large Kenyan dataset that is the focus of this study, we observe that HB expression differs significantly for severe versus mild disease, and for rosetting versus impaired consciousness associated severe disease. The analysis of a second much smaller dataset from Mali suggests that these HB-phenotype associations are consistent across geographically distant populations, since we find evidence suggesting that the same HB-phenotype associations characterize this population as well.

**Conclusions:**

The distinction between rosetting versus impaired consciousness associated *var* genes has not been described previously, and it could have important implications for monitoring, intervention and diagnosis. Moreover, our results have the potential to illuminate the molecular mechanisms underlying the complex spectrum of severe disease phenotypes associated with malaria—an important objective given that only about 1% of *P. falciparum* infections result in severe disease.

## Background

The main target of the human immune response to *P. falciparum* is the antigenic protein *P. falciparum* erythrocyte membrane protein 1 (PfEMP1) [1], which is expressed on the surface of infected red blood cells and serves to bind host endothelial receptors. PfEMP1 is encoded by the members of the hyper-diverse *var* gene family, of which there are about 60 per parasite genome. These genes encode proteins that typically differ at the amino acid level by 34-55% in the extracellular region of the protein that is the most highly conserved [2]. *Var* gene variants switch expression in a mutually exclusive manner over the course of an infection as a means of immune escape. It is thought that different PfEMP1 variants exhibit different binding preferences, which in turn result in different manifestations of disease (reviewed in, e.g., [3]).

Thousands of distinct *var* sequences exist even within small local populations. The sequences that make up an individual parasite’s *var* repertoire typically differ from one another as much as *var* sequences sampled at random from the population, and in many populations there is negligible overlap between individual *var* repertoires [2]. The *var* sequence diversity that exists both within and between genomes is thought to account for the remarkable persistence and recurrence of infections within hosts. Due to variation in the domain composition of *var* genes, and the high levels of sequence diversity within domain families, *var* sequence variants cannot be reliably aligned by traditional methods. However, it is nevertheless clear that *var* diversity arises from a common set of ancient sequence fragments that recombine at exceedingly high rates [4-7]. In line with this, it has been shown that a relatively small set of so-called homology blocks (HBs) can describe ~83% of the *var* sequence diversity found within a set of distantly related parasite genomes originating from diverse locations around the globe [8].

*Var* diversity within local populations is typically analyzed by sampling a ~125aa sequence tag within DBLα subdomain 2 (e.g., [2]). The classic method to distinguish different tag types, which is used in most of the previous studies of *var* diversity (including [9,10]), relies on either the specific amino acid sequence (a level of diversity at which almost all sequences are distinct), or the presence/absence of short perfectly conserved motifs (e.g., the cysPoLV groups and the H3 subset, and when in combination with network based sequence analysis methods, the block-sharing groups that define A-like *var* genes) [11-13]. Some of these classic tag types are thought to be associated with certain disease phenotypes. One relatively consistent finding is that A-like *var* expression is associated with both rosetting [13-15] and severe disease [12], though not necessarily independently since it is well established that the rosetting phenotype correlates with severe disease [16-19]. Rosetting is defined as the binding of uninfected red blood cells by infected red blood cells. This phenotype can be clinically assayed at low cost, and it provides a particularly good starting point to look for genotype-phenotype associations because, rather than being determined by a multitude of parasite and/or host factors, it is thought that rosetting is directly mediated by PfEMP1 binding. Furthermore, the DBLα domain is thought to contain the actual site for PfEMP1 binding of uninfected cells [20], so variation within the DBLα tag may be expected to influence variation in the rosetting phenotype. Severe malaria has also recently been linked to particular domain cassettes that include the DBLα domain [21-24]—a finding that suggests a possible association between DBLα and disease severity, and further increases the likelihood that residues important for disease phenotype exist in the protein region encoded by DBLα tags. All of the above evidence, taken together with the great amounts of DBLα tag data presently available, makes this sequence region very attractive to study.

The most comprehensive DBLα tag dataset currently available was previously analyzed by Warimwe et al. [9,10]. It includes expressed DBLα tags (cDNA) and clinical data for 250 isolates from Kenya, as well as a sample of genomic DBLα tags for 53 isolates. This dataset supports the above mentioned association of A-like *var* expression with both rosetting and severe disease. Warimwe et al. also report another interesting set of patterns within this data: while A-like expression associates with one form of severe disease, impaired consciousness (IC), it does not correlate with another form of severe disease, respiratory distress (RD); additionally, while rosetting correlates with both RD and A-like *var* expression, it does not correlate with IC [10]. Based on these observations, Warimwe et al. conclude that two subsets of A-like *var* genes must exist that cause disease by very different means. They hypothesize that the subset associated with impaired consciousness causes severe disease through tissue specific sequestration, while the subset associated with rosetting causes RD and sometimes also IC through a non-tissue-specific mechanism; however, they were unable to identify a genetic marker that could distinguish these two subsets of *var* genes [10]. One possibility is that the *var* DBLα tag does not contain the differentiating factor, but another possibility is that the methods used by Warimwe et al. to distinguish different types of tag sequences did not fully capture all the functionally relevant genetic variation within the tag.

Here we address whether it is possible to capture more of the phenotypically relevant genetic diversity within a *var* DBLα tag by taking advantage of its homology block architecture. We hypothesize that since HBs are the units of sequence conservation and the means by which diversity is generated in *var* genes (i.e. through recombination), they may reflect functionally relevant sequence diversity that correlates with disease phenotype. To test this hypothesis, we reanalyzed the data originally analyzed by Warimwe et al. [9,10], looking for correlations between the expression of particular homology blocks and the occurrence of particular disease phenotypes. We find that a generic set of HBs, which were defined using only a few geographically distinct isolates [8], are capable of describing the variation observed at this local scale in Kenya. When we test for genotype-phenotype relationships, we find that those described by HBs are statistically stronger than those described previously. We further show that a principal component analysis (PCA) of HB expression rate profiles across isolates can break down HB variation in a way that is useful for generating high quality genotype-phenotype models.

## Methods

### Homology block nomenclature

The DBLα homology blocks discussed here are those described in Rask et al. [8]. These are distinct from the DBLα “homology blocks” of Smith et al. [25] and the DBLα “blocks” of Bull et al. [12] both in definition, and for the most part, in practice. Therefore, wherever we refer to homology blocks (HBs) below, we mean those of Rask *et al*., and we use their system of numbering to refer to particular HBs as well.

### Data and HB assessment of sequences

The expressed sequences and the clinical data for 250 isolates (217 symptomatic, 33 asymptomatic) were obtained from the online supplementary information of [10]. The genomic sequences for 53 isolates were obtained from EMBL using the reference numbers in [9] for the genomic sequences: FN592662–FN594512. The expression rate of classic *var* types, which are defined by presence/absence of specific motifs in the case of cys2PoLV groups and h3sub *var* types, and by network analysis in the case of A-like and BS1/CP6 *var* types, were also obtained from the online supplementary information of [10]. All sequences were analyzed to assess HB composition. HBs were identified using the VarDom Server [8]. A gathering cut-off of 9.97 was used as the threshold to define a match.

### Linkage analysis of HBs in genomic sequences

Linkage analysis was based on the linkage disequilibrium coefficient, D, among HBs within the 53 genomic isolates. The statistical significance for D values is determined by the method described in [26]. Where noted, D is normalized to account for the fact that D is maximized for intermediate frequency HBs (Additional file 1: Figure S3). Normalization is done by dividing D by (pq(1-p)(1-q))^2^, where p and q are the frequencies of the two HBs being analyzed for linkage.

### HB expression rate

The HB expression rate for a given isolate was defined as follows: the number of HBs of a certain type found within the expressed sequences of a given isolate (the expressed sequences consist of each unique expressed sequence represented as many times as it is found within that isolate), divided by the total number of expressed sequences for that isolate.

### Phenotype association networks

For the purposes of creating phenotype association networks, we analyzed the 217 symptomatic isolates within the dataset. For continuous phenotypes, we included in the network any significant correlation or rank correlation between a phenotype and an HB/*var* type expression rate or PC (p ≤ 0.05). For binary phenotypes, we included all associations where the mean expression rate or PC was found to be significantly different for the two phenotypic states (p ≤ 0.05 by Friedman Rank, Kruskal-Wallis and/or K-Sample T, where each test is applied only when appropriate). HBs that are linked to similar phenotypes can be defined by analyzing networks in which HBs are connected by edges to the phenotypes with which their expression is correlated. We do not correct for multiple hypothesis tests in determining these edges because the conclusions are based on the consideration of many edges taken together, and a more lenient threshold allows the network to capture a greater number of meaningful biological signals.

### Transformation of expression rates and rosetting level

Prior to performing all linear and logistic regression analyses, the expression rates for particular *var* types (i.e., cys2, A-like, group 1, group 2, group 3, BS1/CP6 and H3sub *var* genes), the HB expression rates (i.e. for all 29 HBs), and the rosetting rates were transformed as described in [10]. The transformation (which is an arcsine transformation with special treatment for extreme values) is a standard method, and makes the data appropriate for fitting with regression models.

### Principal component analysis

A PCA was carried out on a dataset of the HB expression rate profiles for the 217 symptomatic isolates. The expression rate profile is the set of expression rates for all 29 HBs for a given isolate. A PCA defines differentially expressed HB components—i.e., orthogonal principal components (PCs). Network analyses and phenotype correlation tests were then carried out using these PCs as independent variables. To test the robustness of the PCA results, we repeated the PCA using non-overlapping subsets of isolates.

### Modeling genotype-phenotype associations

Phenotype correlation tests consisted of multiple linear and logistic regression models, similar to the tests performed in [10], however in our case we substituted the expression rates of classic *var* types for HB expression rates, or PCs of HB expression rate profiles. BIC, AIC, R^2^ and Adjusted R^2^ were all used to compare the quality of alternative models. Where indicated, host age was included as an independent variable even where it did not appear to have a significant effect in order to eliminate the potential for observing spurious correlations resulting from co-correlation with this variable, since many weak correlations between disease phenotype and host age have been reported previously (e.g., [27]).

### Variable selection to optimize models of rosetting

To select a set of independent variables that produce the most informative model of rosetting, we started with many possible independent variables in a multiple linear regression model, and then successively removed the least significant contributing variable, excluding host age, until the BIC stopped decreasing. We then verified that the BIC increased with the removal of any of the final independent genetic variables. The BIC, AIC, R^2^ and adjusted R^2^ scores for the final models after removing host age were also evaluated. Most variable selection procedures were also carried out under the scenario where host age is removed as soon as it is the least significant contributing variable, and in all cases examined this had no influence on the variable selection results.

### Identifying rosetting associated HBs or PCs

Warimwe et al. test whether particular expression rates can significantly reduce the explanatory power of rosetting on RD as a means to identify a group of *var* genes that associate with rosetting and RD as opposed to impaired consciousness [10]. However, we reason that even a perfect genetic marker may not substantially reduce the effect of the rosetting coefficient. If there is a tighter relationship between rosetting and RD than between the expression rate of the responsible gene and RD (which is likely the case if the path from gene to rosetting to RD accumulates noise along the way), then the most informative regression model will still primarily depend on rosetting as the primary independent variable. For this reason we take a different approach. We attempt to identify rosetting-specific *var*/HB expression rates or PCs by considering which *var*/HB expression rates or PCs remain as independent predictive variables in a model of rosetting after the variable selection procedure described above.

## Results and discussion

### Using HBs to classify var types within a local population

Many of the HBs identified in this dataset were also found in the genome of the chimpanzee malaria parasite *P. reichenowi* (HBs 5, 14, 36, 64, 54, 60, 79, 210, 88, 131, 153, 171, 163, and 260, in order of frequency in the *P. reichenowi* genome). Sequence homology among such distantly related parasites reflects the ancient origin of *var* genes, and the strong balancing selection that maintains these sequence variants through millions of years of evolution [28].

**Figure 1:**
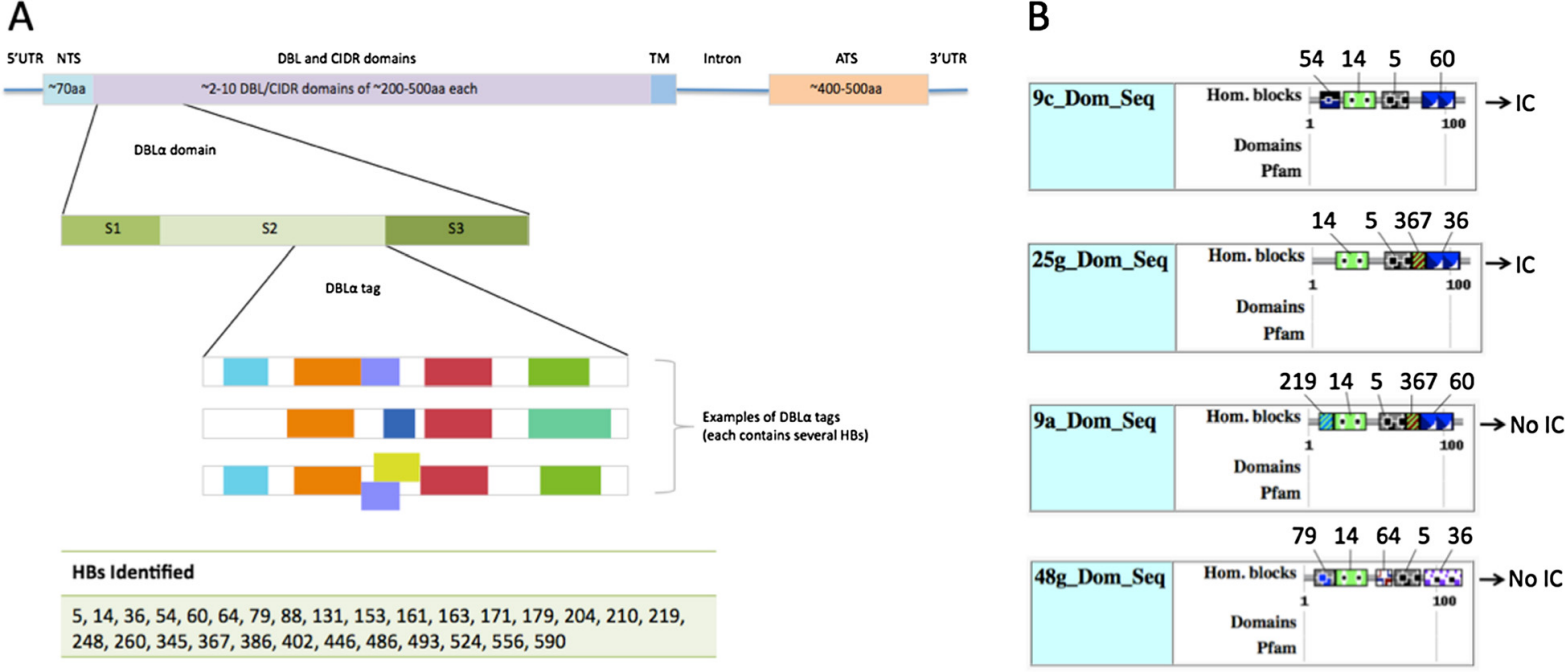
**The homology block architecture of DBLα tags. (A)** The architecture of a *var* gene and the PfEMP1 protein it encodes. The number, identity and order of the DBL and CIDR domains varies. One of the only constants is the presence of a single DBLα domain, which is located at the N-terminal end of the coding region. The DBLα domain is made up of subdomains S1-3. The tag comes from a region of S2. Twenty-nine distinct homology blocks were found within the cDNA dataset and almost the same set (all but HB 556) were found within the genomic dataset. **(B)** The output from Vardom Server [8] with added HB labels for the dominantly expressed sequence tags for four of the highest rosetting isolates within the cDNA dataset, chosen as follows: from the symptomatic isolates with the highest rosetting rates (i.e., the 22 isolates with transformed rosetting rates over 0.5), we identified those with a single dominantly expressed sequence (i.e., approximately twice as large as the expression rate of any other sequence or more, and larger than the rest of the sequences’ expression rates combined), and this amounted to seven sequences; the four shown are those with good HB coverage (more than 3 HBs within the tag). It is indicated whether the patient from which the sample was taken exhibited impaired consciousness (IC).

The genomic *var* dataset, comprising 1851 sequences, contained 1708 unique sequences by amino acid identity (aa-types), with an average of 34.92 aa-types per isolate. There were 2–10 HBs per DBLα tag (Figure 1), and the genomic dataset contained 28 unique HBs in 398 unique combinations (398 HB-types), with an average of 5.19 HB-types per isolate. The cDNA dataset for all 250 isolates, comprising 4538 sequences, contained 3925 unique sequences by amino acid identity, with an average of 18.15 aa-types per isolate. These sequences contained 29 HBs in 557 unique combinations, with an average of 2.23 HB-types per isolate.

**Figure 2:**
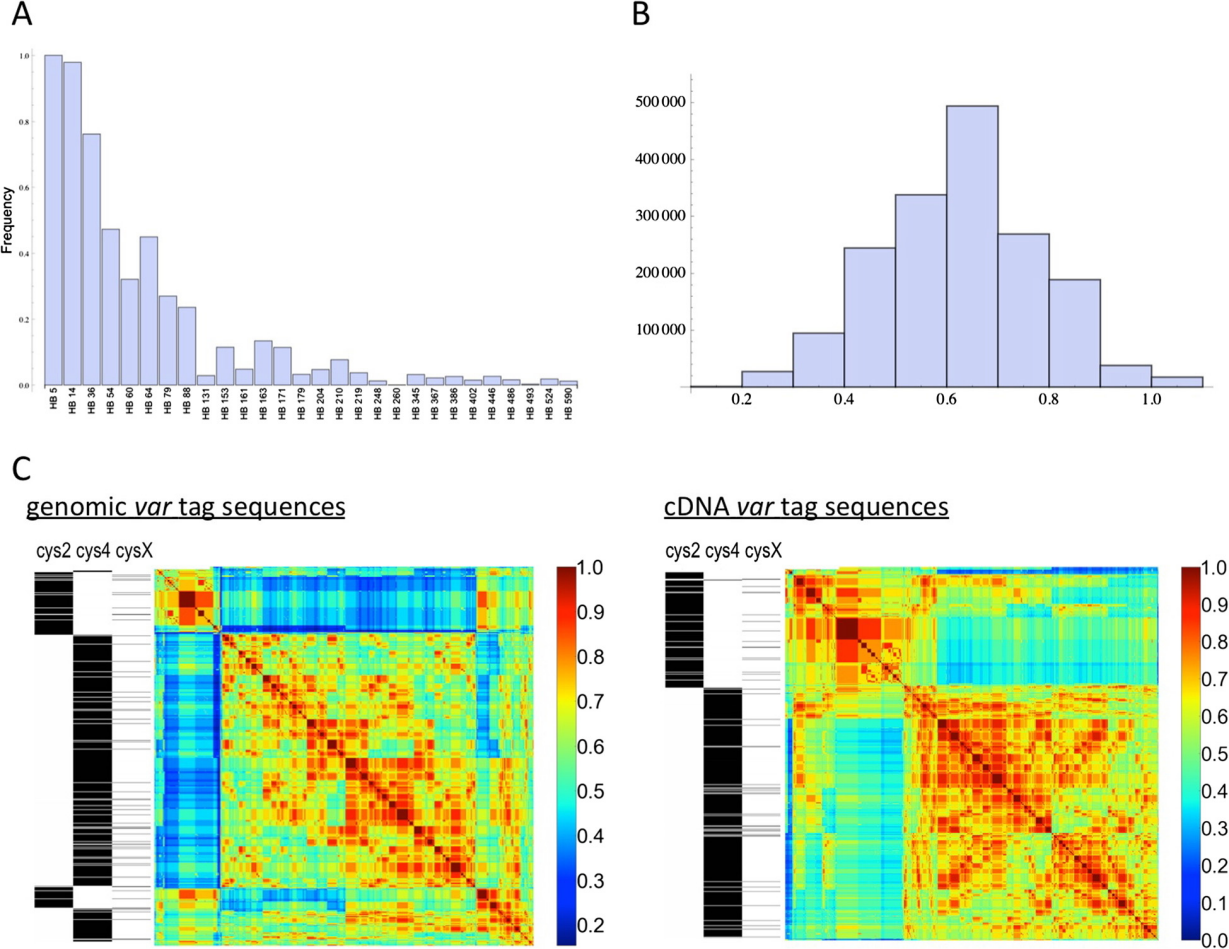
***Var *diversity within a local population is captured by homology blocks. (A)** Frequency of each HB in the dataset of genomic *var* tags. **(B-C)** The pairwise similarity among sequence types, where types are defined by homology block composition: the number of HBs shared between any two sequences divided by the average number of HBs within a sequence for those two sequences. **(B)** Frequency distribution of pairwise HB similarities between sequences in the genomic dataset. The approximately normal distribution contrasts with the bimodal distribution that has been observed for other data, when pairwise similarity is defined by amino acid identity [29]. **(C)** Sequences are hierarchically ordered based on pairwise HB similarity using the average-linkage method as implemented in SciPy. The distinction between sequence tags containing two cysteines (cys2) versus four (cys4) is very clear, reflecting that recombination occurs at a faster rate within, relative to between, the two groups.

For the dataset of cDNA *var* tags for all 250 isolates, the average fraction of the sequence that is missed by HB alignment is 12.7% (when the sites before the start of the first HB and after the end of the final HB are excluded). The frequency of the HBs varied, with only a few at intermediate frequencies (Figure 2A). The sequences were highly variable in their HB composition (Figure 2B), and reflected the previously described recombining groups (Figure 2C).

**Figure 3:**
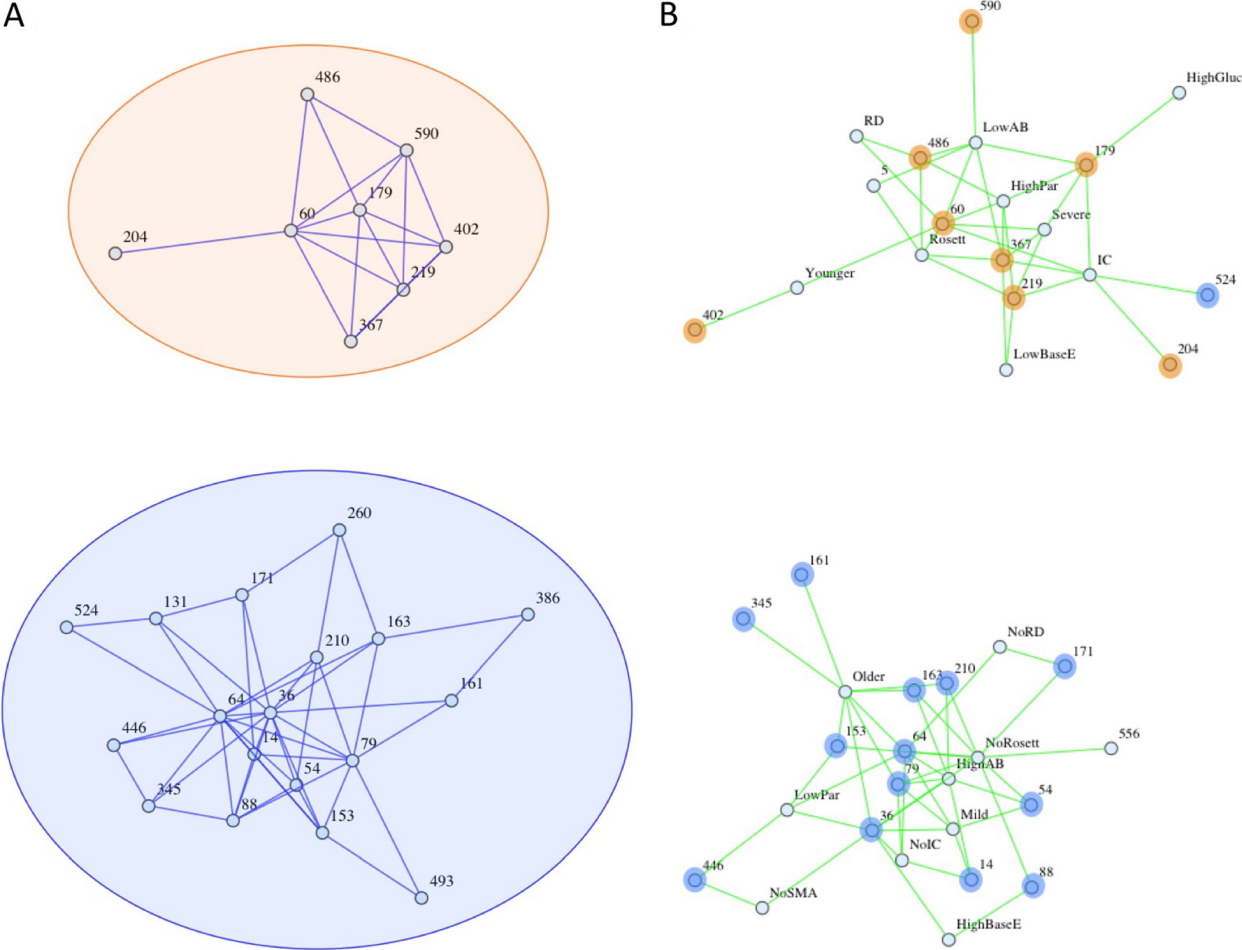
**Two HB subnetworks: associated with severe versus mild spectrum disease.** HB networks reveal two discrete HB subsets—one being associated with severe spectrum phenotypes (orange) and the other being associated with mild spectrum phenotypes (blue). **(A)** The network of significant positive linkage disequilibrium coefficients (D) among HBs in the genomic dataset, based on a one-tailed significance threshold of p ≤ .025, reveals two subnetworks of linked HBs. **(B)** The network of significant associations between HB expression rates and phenotypes (p ≤ 0.05) with nodes colored according to the subnetworks of A. The HBs in the orange subnetwork are generally associated with severe disease spectrum phenotypes, whereas those in the blue subnetwork are generally associated with mild. The lack of connectivity between the severe and mild spectrum phenotypes in A is highly significant: even just considering the nodes of degree 3 or less, p < 0.0001 for the fact that each HB in the network is associated with mild or severe spectrum phenotypes, but not both. SMA = severe malarial anemia, Rosett = rosetting, RD = respiratory distress, Severe = severe disease, Mild = mild disease, Older = high host age, Younger = low host age, Par = parasitemia, BGlu = blood glucose (low levels indicate hypoglycemia), BaseE = base excess (low levels indicate metabolic acidosis), AB = antibody response.

While the diversity of HB-types is almost an order of magnitude less complex than the diversity of aa-types, the former is nevertheless considerable and potentially functionally informative (Figure 3). Thus, even though these HBs were designed with reference to the *var* diversity of only a few parasite genomes (i.e., those analyzed in [8]), most of the sequence variation present within this local population is captured by homology to HBs, and so it is reasonable to hypothesize that the HBs capture functional variation among DBLα tags in this population, at least with regard to phenotypes known to be mediated by the DBLα domain. For example, it seems reasonable that the unique aspects of the HB composition observed for rosetting associated *var* tags (Figure 1B; Additional file 1: Figure S2) may be of functional significance.

### Defining groups of associated HBs through linkage or phenotype correlation networks

With genomic samples, groups of HBs can be defined based on analyzing genomic *var* diversity through a simple linkage analysis of the positive linkage disequilibrium coefficient (D) values that exceed a one-tailed significance threshold of p ≤ .025 [26]. The observed number of positive pairwise linkages that lie beyond this 95% confidence interval is 65, which greatly exceeds the expected number under the null hypothesis of random associations, 9.45. The presence of significant linkages among HBs implies that sequences are not random sets of HBs even after taking into consideration the observed HB frequencies. The weighted network of linkages among HBs (the positive normalized D values, significant and non-significant) can be analyzed for community structure (Additional file 1: Figures S3 and S4), and we find that the two communities that result from this analysis agree exactly with the two subnetworks of HBs described by the significant linkages among HBs (Figure 3A).

Using expression data, we can measure the expression rate for each HB in each isolate, and we observe many correlations among HB expression rates (Additional file 1: Figure S5). HB expression data also reveal that the two linkage groups of HBs are associated with very different manifestations of disease. With the observed correlations between HB expression rates and disease phenotypes we can build a network of significant associations between HBs and phenotypes, and define groups of HBs based on their associations with similar phenotypes. We find that two primary groups of HBs emerge from this phenotype association network (Figure 3B), and they correspond to the two groups defined by HB linkage within genomic sequences. This correspondence between the linkage and phenotype association subnetworks supports the idea that HBs may be able to serve as robust markers for functional differences among *var* genes.

### Distinguishing two subsets of A-like var tags with different phenotype correlations

**Figure 4:**
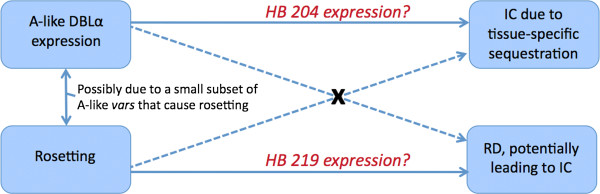
**Two subsets of A-like *var *genes differently associated with severe disease.** Prior analyses by Warimwe et al. [10] established that while A-like expression associates with one form of severe disease: impaired consciousness (IC), it does not correlate with another form of severe disease: respiratory distress (RD). Furthermore, while the rosetting phenotype (which correlates with A-like *var* expression) was found to associates with RD, it was not found to associate with IC. Warimwe et al. concluded that there must be two subsets of A-like *var* genes that cause severe disease by distinct means: one that causes impaired consciousness by tissue-specific sequestration, and another that causes rosetting, which can lead to respiratory distress (RD). HBs—particularly HBs 204 and 219—improve our ability to distinguish these two classes of severe spectrum *var* genes.

Earlier analysis of the data by Warimwe et al. established that, while A-like *var* expression is associated with rosetting, A-like *var* expression and rosetting appear to be independent with regard to their associations with disease phenotypes. Specifically, while A-like *var* expression is correlated with impaired consciousness but not respiratory distress, rosetting is correlated with respiratory distress but not impaired consciousness [10]. This observation led Warimwe et al. to conclude that there must be a small subset of A-like *var* genes that cause severe disease through a specific rosetting-dependent mechanism (Figure 4). However, their methods—which rely the expression rates of classic *var* types—did not reveal any statistically significant support for the existence of such a subset [10] (Additional file 2). By classic *var* types we henceforth mean the seven that are examined in this prior analysis: cys2, A-like, the H3 subset (h3sub), cysPoLV groups 1, 2, and 3, and BS1/CP6 [10].

In an attempt to identify this hypothesized class of *var* genes using HBs, we looked for a subset of A-like *var* genes that have expression rates significantly correlated with rosetting, and simultaneously significantly anti-correlated with IC. Among the expression rates of classic *var* types, none had significant and opposite associations with rosetting and IC. Among the HB expression rates we tested, there were many with significant associations with rosetting (data not shown, but see Additional file 1: Figure S9 ) and/or IC (Additional file 1: Figure S8), but only one had significant associations with these phenotypes in opposite directions: The expression rate of HB 204 is significantly anti-correlated with rosetting (p = 0.025) and significantly correlated with IC (p = 0.0069) in models using HB 204 and host age as the only independent variables (Additional file 1: Figure S8).

Next we addressed whether any HBs can provide additional information about rosetting, beyond what is already captured by classic *var* tag typing methods. We added each HB expression rate as an additional independent variable, one at a time, into a model of rosetting that already contained eight other independent variables: host age and the expression rates for the classic *var* types. We then compared model statistics (primarily BIC, but also AIC, R^2^ and adjusted R^2^) to determine the benefit of the particular HB expression rate to the model (Additional file 3: Table S1). While most HBs increase the BIC, decrease the adjusted R^2^ and provide an insignificant contribution to predicting rosetting (p>> 0.05), two HBs make improvements to the model and have significant p-values even within these over-parameterized models. HB 204 substantially reduces the BIC (from 50.72 down to 48.62), and substantially increases the adjusted R^2^ (from 0.348 up to 0.376). HB 54 is the only other HB to reduce the BIC and increase the adjusted R^2^ of the original model, however it only brings the BIC down slightly (to 50.65) and the adjusted R^2^ up slightly (to 0.367) (Additional file 3: Table S1).

### Variable selection to achieve a model of rosetting

In order to identify what genetic variation best explains the variation observed in rosetting, we performed a variable selection procedure to find the optimal set of independent variables for a multiple regression model of rosetting. Three tests were performed, which together show that HB 219 is a better predictor of rosetting than any of the classic *var* types (Table 1):

**Table 1:** Statistics for multiple regression models predicting rosetting*

	**Independent variables**	**AIC**	**BIC**	**R2**	**Adj. R2**
**A**	**Cys2**, Grp2, Grp3, **BS1CP6**	20.14	37.40	0.358	0.338
**B**	HB36, HB204, HB210, **HB219**, **HB486**	16.48	36.60	0.385	0.361
**C**	**BS1CP6**, HB54, HB171, HB204, **HB219**	14.02	34.14	0.400	0.373
**D**	**BS1CP6, PC1, PC3, PC4, PC22**	4.776	24.90	0.438	0.415

*The result of removing the least significant genetic variable, one by one, from models of rosetting that start with the expression rates of: (row A) the 7 classic *var* types, (row B) the 29 HB expression rates, (row C) the expression rates for both the 7 classic *var* types and the 29 HBs, and (row D) the expression rates for the 7 classic *var* types and the 29 PCs. The variable selection procedure is done maintaining host age in the model, however statistics are shown with age removed. Positive effect independent variables are shown in boldface.

In a first test, we start with a model that initially includes all seven classic *var* types plus host age. We successively remove the genetic variable that contributes least significantly to the model until the BIC and related statistics are optimized (see Methods for details). We find that the model with the lowest BIC contains the expression rates for cys2 and BS1/CP6 *var* types as positive predictors of rosetting, and the expression rates for cysPoLV group 2 and cysPoLV group 3 *var* types as negative predictors of rosetting (BIC = 37.40) (row A in Table 1 and Additional file 3: Table S3).

In a second test we start with all 29 HB expression rates plus host age as independent variables and then we follow the same variable selection procedure. In this case the resulting model is one with HB 36, HB 204 and HB 210 as negative predictors of rosetting, and HB 219 and HB 486 as positive predictors of rosetting (BIC = 36.60) (row B in Table 1 and Additional file 3: Table S3).

In a third variable selection test we start with all 29 HB expression rates in addition to the expression rates for all seven classic *var* types, plus host age. Starting with this initial set of independent variables, the model that results after variable selection is one containing the expression rates of BS1/CP6 and HB 219 as positive predictors of rosetting, and the expression rates of HB 54, HB 171 and HB 204 as negative predictors of rosetting (BIC = 34.14) (row C in Table 1 and Additional file 3: Table S3; Additional file 1: Figure S10).

Two additional anecdotes provide further credibility to our finding that HB 219 expression rate is a robust positive predictor of rosetting: First, we find that in all of the nine cases where there is rosettting data for an isolate that has HB 219 present in its most highly expressed sequence, considerable rosetting is observed (defined as > 0.1). Secondly, we find that the DBLα domains of known rosetting *var* genes [30,31] contain HB 219 (Additional file 1: Figure S2).

Based on a comparison of the BIC scores of the models that result from the above variable selection procedures (Table 1), it seems that a more informative model for rosetting can be achieved when HB expression rates are used as candidate independent variables in addition to classic *var* types. More specifically, the most informative model is achieved when we consider the expression rates of several HBs in addition to the expression rates of one classic *var* type: BS1/CP6. This becomes even clearer when we perform a fourth variable selection procedure using the principal components discussed below (row D in Table 1 and Additional file 3: Table S3).

### Principal components of HB expression rate profiles and variation in rosetting

We perform a PCA on the HB expression rate profile, which we define as the set of expression rates for all 29 HBs. This deconstructs the HB expression rate profiles into orthogonal principal components (PCs) based on how they vary across different isolates. We then repeat the above network and variable selection analyses using PCs in place of individual HB expression rates (Additional file 1: Figures S11 and S12).

**Figure 5:**
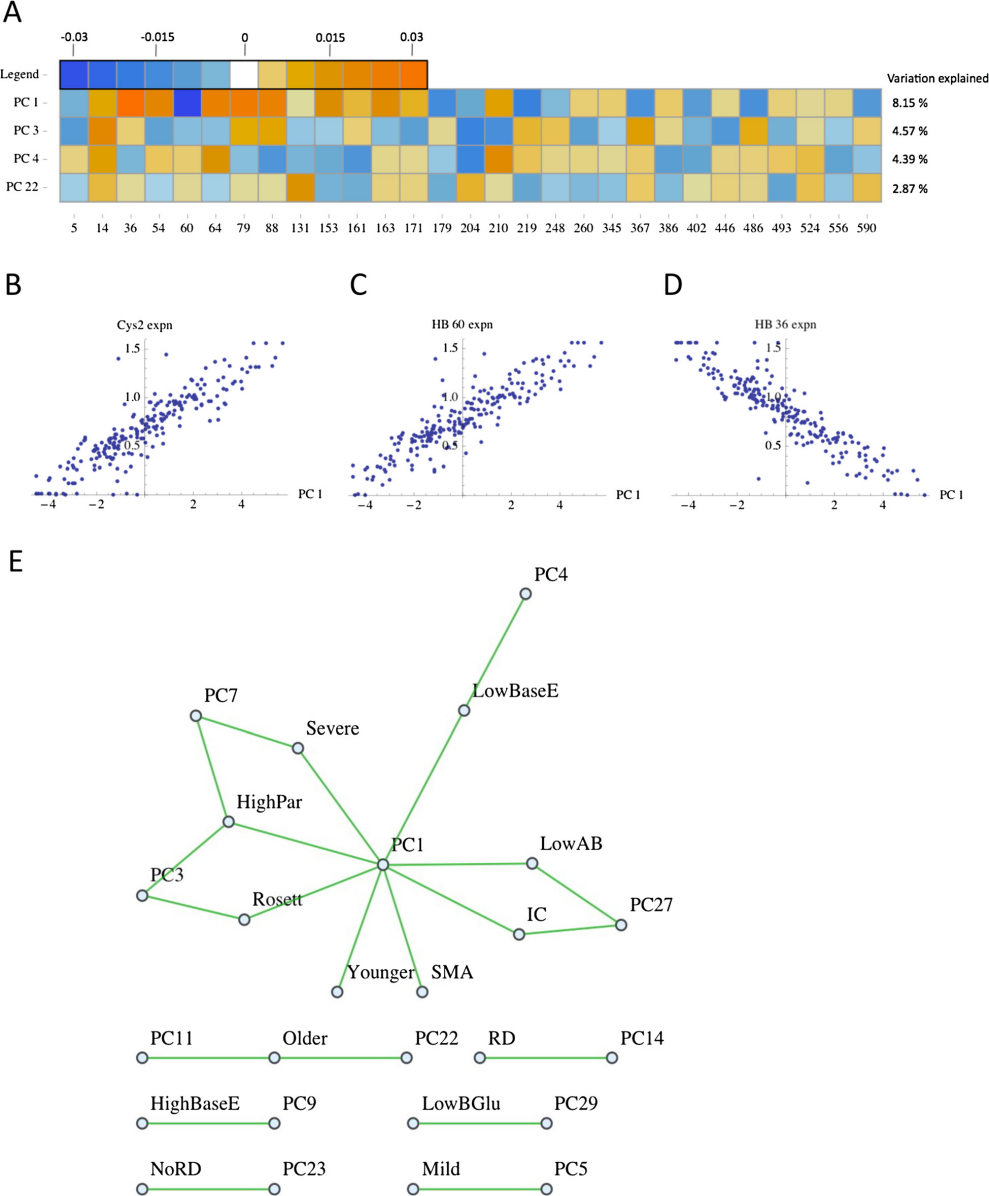
**Principal components of HB expression rate profiles. (A)** PCs expressed as coordinates in the original space of the data, which is the expression rates for all 29 HBs for each of the isolates. The amount of variation among the isolates that is explained by each of the PCs is shown on the right. **(B)** The PCA of HB expression rate profiles reflects the differentially expressed HB components, and the first PC defines the extent to which there is a bias toward the expression of *var* tags with 2 cysteines (cys2). The cys2 expression bias maps roughly to an association with mild versus severe disease spectrum phenotypes. **(C)** PC1 (and Cys2 *var* gene expression) correlates with the expression of several HBs, including HB 60. **(D)** PC1 (and Cys2 *var* gene expression) anti-correlates with the expression of several HBs, including HB 36. **(E)** The network of significant correlations between HB expression rate profile principal components (PCs) and disease phenotypes (p ≤ 0.05). SMA = severe malarial anemia, Rosett = rosetting, RD = respiratory distress, Severe = severe disease, Mild = mild disease, Older = high host age, Younger = low host age, Par = parasitemia, BGlu = blood glucose (low levels indicate hypoglycemia), BaseE = base excess (low levels indicate metabolic acidosis), AB = antibody response.

We find that PC 1 is related to the cys2 versus non-cys2 distinction (Figure 5B), and that it captures the difference between HBs that are associated with severe versus mild spectrum phenotypes (Figure 3; Additional file 1: Figure S4). PC 1 correlates with all of the severe spectrum phenotypes (Figure 5E) and the HB expression rates that contribute most to PC 1 are those with strong associations with disease phenotypes. PC 1 describes 8.15% of the variation among isolates with regard to their HB expression rates (Additional file 1: Figure S14). The HBs that have large positive values in PC 1 define the core of the mild spectrum linkage/phenotype subnetwork (Figures 3, 5A and D; Additional file 1: Figures S4 and S13). Likewise, the HB that has the dominant negative value in PC 1, HB 60, defines the core of the severe spectrum linkage/phenotype subnetwork (Figures 3, 5A and C; Additional file 1: Figures S4 and S13). These observations about PC 1 are robust to the specific isolates used for the PCA. When non-overlapping subsets of isolates are analyzed separately, the relative contributions of the various HB expression rates that primarily contribute to PC 1 remain essentially the same (Additional file 1: Figure S15).

We address whether the PCs provide additional information about rosetting beyond what can be predicted based on the expression rates of the classic *var* types. We start with a multiple regression model of rosetting that has the seven classic *var* types, plus host age, as independent variables. We then add each of the PCs, one at a time, and observe whether they make a significant contribution to predicting rosetting and/or reduce the BIC of the model. The only PC that is significantly predictive about rosetting in the context of this already over-parameterized model is PC 3, which shows a positive association with rosetting. PC 3 is also the only PC to reduce the BIC (from 50.72 down to 48.36), and it also reduces the AIC (from 21.97 down to 16.73) and increases the adjusted R^2^ (from 0.348 to 0.378) (Additional file 3: Table S2).

The above findings suggest that, regarding the rosetting pattern, PC 3 provides qualitatively different information from any of the classic *var* types. PC 3 is dominated by a strong negative value in the dimension of HB 204 expression rate (Figure 5A), which is consistent with PC 3 having a positive association with rosetting, since we established above that HB 204 significantly anti-correlates with rosetting.

Next we perform a variable selection procedure to address whether an optimized model of rosetting will contain PCs or classic *var* types, or both. We start with a multiple regression model of rosetting that includes all 29 PCs and all seven classic *var* types, and host age, as the independent variables. We follow the variable selection procedure (described in Methods), and we find that the most informative model by BIC includes the following genetic variables: BS1/CP6, PC 1, PC 3, PC 4 and PC 22 (row D in Table 1 and Additional file 3: Table S3) (BIC=24.90).

The PC-containing models have much lower BIC scores and higher adjusted R^2^ values compared to all other models (row D in Table 1 and Additional file 3: Table S3). This means that the PCA is able to consolidate the relevant functional variation into fewer variables by replacing a handful of HB expression rates with a single PC and still retaining the same ability to predict rosetting. For example, relative to any individual expression rate, PC 1 appears to be a better predictor of whether an isolate will express severe spectrum phenotypes or mild spectrum phenotypes. Thus, the expression rates of many HBs appear to be non-independent with respect to their relationships to phenotype. Our PCA results also imply that within the small DBLα tag there are multiple independent genetic components that are relevant to disease phenotype, since otherwise we would not expect to find more than one PC playing a significant role in any of the phenotype prediction models. This conclusion is consistent with the fact that many of the first several PCs explain similar levels of variation among isolates (Additional file 1: Figure S13 and S14).

The principal components improve phenotype prediction, but they are less straightforward to interpret than individual HB expression rates. Nevertheless, our results demonstrate that PC 1 clearly corresponds to the major division found by network analyses, severe and mild spectrum associated *var* genes.

Furthermore, the various correlations between phenotypes and PCs, and between the expression rate of various sequence types and PCs, can be summarized in networks, which can provide additional means to interpret the PCs (Figure 5E; Additional file 1: Figure S11). In summary, we find that two PCs capture interesting phenotypic distinctions among isolates, and we find that model BICs improve considerably when PCs are used in place of individual HB expression rates.

### The consistency of HB-phenotype associations in distinct populations

HB analysis of a smaller dataset from Mali that was originally analyzed by Kyriacou et al. [14], reveals that at least some of the HB-phenotype associations reported above are similarly informative in geographically distinct (and presumably genetically unrelated) populations. Twenty-four of the 29 HBs we identified in the Kenyan dataset (Figure 1) were present in the Malain dataset (data not shown). The Malian dataset contains 9 isolates from cerebral cases of malaria, and 8 isolates that serve as negative control for severe disease since they are from mild hyperparasitemic cases. Kyriacou et al. argue that mild hyperparasitemic malaria is the appropriate negative control for cerebral malaria since the two forms of disease exhibit comparable levels of parasitemia. Given the null hypothesis that the probability of expressing a certain *var* type is the same in both the mild hyperparasitaemic malaria patients and cerebral malaria patients, we calculate the probability (by Fisher’s exact test) of the data observed by Kyriacou et al., and we find that the distribution of HB 36 is less likely than the distribution of cys2—indicating that HB 36 is a stronger marker of severe disease than cys2 in the Malian population. This is essentially what we observed in the Kenyan population, since HB 36 is the dominant HB expression rate of the PC that correlates most strongly with severe disease, PC 1 (Figure 5E). Additionally, in the Malian population we find that HBs 60, 64, 79, 163, and 179 are differentially expressed in cerebral versus mild hyperparasitaemic cases (p < .05).

For the Malian dataset [14], we also compare the recall (hit rate), accuracy and precision of the following two predictive models: (1) expressed DBLα sequence tags containing two cysteines predict severe malaria whereas those with some other number predict mild hyperparasitaemic malaria, and (2) expressed sequence tags lacking HB 36 predict severe malaria whereas those with HB 36 predict mild disease. The hit rate, accuracy and precision are given by TP/P, (TP + TN)/(P + N) and TP/(TP + FP), respectively, where TP is the number of truly positive instances classified as positive, TN is the number of truly negative instances classified as negative, FP is the number of truly negative instances classified as positive, P is the total number of truly positive instances classified as either positive or negative, and N is the total number of truly negative instances classified as either positive or negative [32]. For the purpose of predicting severe disease from sequence features of expressed DBLα *var* tags in the Malian population, classification by HB 36 out-performs classification by cys2 in terms of all three of the above. The hit rate is 0.723 as opposed to 0.617, the accuracy is 0.765 as opposed to 0.724, and the precision is 0.773 as opposed to 0.763.

Among the unique set of sequences expressed within the cerebral and hyperparasitemia isolates, the rank correlations (both Spearman and Kendall) of rosetting with each of HB 60, 79, 153, and 219 are all greater in magnitude than the rank correlation of rosetting with cys2. These several HBs are also associated with rosetting in the Kenyan dataset [10], and thus, they appear to serve as more informative predictors of rosetting than the number of cysteines within the *var* DBLα tag.

## Conclusions

Even though the HBs were designed using a very small number of *var* sequences isolated from a few parasite genomes, they manage to cover the sequence diversity of a local population, leaving only the minority of sites unaligned. We find that the variation described by HB diversity within the *var* DBLα tag is not completely redundant with the diversity already described by classic methods. Furthermore, relative to classic methods, the consideration of HB composition appears to be more informative for predicting whether a tag’s expression is associated with various disease phenotypes.

All of the HBs within the optimized rosetting model (HBs 171, 204, 54 and 219; row C in Table 1) are located at the N-terminal end of the tag (Additional file 1: Figure S16). They are also overlapping with the PoLV1 site (position 3–5 in each of the above HBs), which distinguishes cysPoLV group 1 *var* genes from other cys2 *var* genes. Based on the defining HMM for HB 204 (Additional file 1: Figure S16) and the definition of cysPoLV group 1, it is clear that HB 204 expression should anti-correlate with cysPoLV group 1 expression, and indeed it does (Additional file 1: Figure S17). From the network analyses (Figure 3; Additional file 1: Figure S4) it can be seen that HB 54 and HB 171 are in the mild spectrum subnetwork, and HB 219 and HB 204 are in the severe spectrum subnetwork. Therefore, HB 204 is unusual in that it maps to the severe spectrum subnetwork, but nevertheless anti-correlates with rosetting. No other HB or classic *var* type shows this pattern, reflecting the fact HB 204 contains unique information that is potentially useful for refining our understanding of the different mechanisms underlying severe disease.

HB 204 expression rate is a significant negative predictor of rosetting regardless of the details of the model. However, its expression is positively correlated with the expression of cysPoLV group 2 tags (correlation coefficient = 0.434, p < 10^-10^), which are by definition cys2. CysPoLV group 2 *var* expression does not predict rosetting in this dataset, either positive or negatively—so possibly HB 204 marks a subset of group 2 *var* genes that do not cause rosetting but that nevertheless cause severe disease, since HB 204 expression is significantly associated with impaired consciousness (however, it is worth noting that HB 204 is also found in *var* genes other than cysPoLV group 2). A final interesting anecdote about HB 204 is that it is part of domain cassette 17 of IT4var13, which is one of the sequence variants known to mediate binding to brain endothelial cells [21].

Warimwe et al. put forward the hypothesis that there are at least two classes of A-like *var* genes: those that cause rosetting and that can lead to RD in severe cases, and those that cause impaired consciousness through a tissue-specific mechanism that does not rely on rosetting (Figure 4) [10]. HB 204 may therefore serve as an ideal marker to distinguish between these two types of severe spectrum genes. Its absence, particularly in the cys2 context, indicates the rosetting phenotype. Its presence marks low rosetting *var* genes that are nevertheless associated with severe disease by way of impaired consciousness.

HB 219 is also interesting because, while its expression is correlated with cysPoLV group 1 expression (Additional file 1: Figures S16 and S17), its expression is more tightly associated with rosetting than cysPoLV group 1 expression is. This is evident in the coefficients and statistical significance of the regression models for rosetting (data not shown), and in the fact that HB 219 expression is an independent variable within the most informative models for rosetting while cysPoLV group 1 expression falls out (Table 1). Since the sequence covered by HB 219 is considerably longer than the MFK motif that defines cysPoLV group 1 *var* genes, it is likely that HB 219 covers additional sequence variation that is either directly or indirectly linked to the rosetting phenotype. Furthermore, HB 219 expression correlates with both high parasitemia and hypoglycemia (Figure 3B). Both of these associations further support the hypothesis that HB 219 is linked to a form of severe disease that manifests through overall high parasite burden rather than through tissue-specific sequestration.

Within the Kenyan population that is the focus of this study, HB expression rates (and to an even greater extent, PCs of HB expression rate profiles) improve our ability to differentiate mild versus severe spectrum *var* genes beyond what is possible with classic typing methods. Furthermore, HBs appear to be informative markers of disease phenotype in more than just this particular population. In a dataset from Mali we again find that HB 219 expression is significantly associated with high levels of rosetting, and that the HB composition of the expressed *var* sequence tags—particularly with respect to HB 36—predicts disease severity with higher precision, accuracy and recall than classic methods. These results suggest that the DBLα HB-phenotype associations, which we characterized using the large Kenyan dataset, are consistent across distinct populations. Thus, a single set of DBLα HBs can potentially serve as parasite genetic markers for severe disease phenotypes in geographically diverse populations. Moreover, the fact that many of the same HB-phenotype relationships are found in two geographically distant populations supports the idea that there is a functional link between particular DBLα HBs and the molecular mechanisms underlying severe disease, since otherwise we would expect recombination to alter HB-phenotype linkages.

In summary, HB typing methods allow for the construction of more specific genotype-phenotype models that in turn suggest that two distinct molecular mechanisms underlie severe malaria. Specifically, we find that *var* DBLα HB 204 expression predicts a form of severe disease that is associated with impaired consciousness and the absence of rosetting, and that *var* DBLα HB 219 expression predicts a form of severe disease that is associated with high rosetting. Insights into genotype-phenotype associations within this system can potentially aid in the development of new diagnostic and monitoring tools for malaria, and perhaps even future vaccines, since *var* genes have been implicated as possible future vaccine targets [33]. Furthermore, if additional studies are undertaken that assess both *var* expression and clinical symptoms, it should be possible to further refine our descriptions of these genotype-phenotype relationships.

Lastly, HBs have the potential to elucidate complex ecological and evolutionary dynamics that potentially shape antigenic diversity within *P. falciparum* populations (e.g., [34]). For example, the fact that the same conserved set of HBs can describe *var* sequence diversity at multiple geographic scales and locations reveals strong balancing selection to maintain ancient sequence fragments across vast expanses of time and space. The complex ecological and evolutionary dynamics that are at play warrant further study because they likely shape *P. falciparum* antigenic diversity, and in so doing, strongly impact the epidemiology of malaria.

## Competing interests

The authors declare no competing interests.

## Authors’ contributions

MMR conceived of the study, carried out the analysis and wrote the manuscript. KPD, MP and TSR contributed to the study design and critically revised the manuscript. EBB contributed to the data analysis and critically revised the manuscript. All authors read and approved the final manuscript.

## Supplementary Material

Additional file 1 **Additional figures. Figure S1.** Respiratory distress (RD) as a function of host age and rosetting. **Figure S2.** HB composition of known rosetting *var* genes. **Figure S3.** Linkage disequilibrium coefficient (D) values for all pairs of HBs in the genomic dataset. **Figure S4.** Community partition of weighted linkage network of HBs. **Figure S5.** HB-HB expression rate correlation matrix. **Figure S6.** Model of respiratory distress. **Figure S7.** Relationship between rosetting and respiratory distress. **Figure S8.** Relationship between impaired consciousness and the expression of various *var* types and HBs. **Figure S9.** The best fit relationship between six variables and rosetting using a window analysis. **Figure S10.** Relationship between rosetting and expression rates of *var* types and HBs. **Figure S11.** PC-classic *var* type association network. **Figure S12.** PC-HB relationships. **Figure S13.** Principal components in data space. **Figure S14.** The amount of variation explained by each PC. **Figure S15.** PCA for two subsets of the data. **Figure S16.** Representation of select homology blocks. **Figure S17.** HB-classic *var* type association network. Click here for file

Additional file 2 Further explanation of methods. Click here for file

Additional file 3 **Additional tables. Table S1.** Multiple regression models of rosetting that include an HB expression rate as an independent variable. **Table S2.** Multiple regression models of rosetting that include an HB expression PC as an independent variable. **Table S3.** Statistics for multiple regression models predicting rosetting with and without age. Click here for file
