# Supplementary material for: Homology blocks of Plasmodium falciparum var genes and clinically distinct forms of severe malaria in a local population
Source: BMC Microbiol. 2013 Nov 6;13:244. doi: 10.1186/1471-2180-13-244 (PMC3827005; doi:10.1186/1471-2180-13-244)
Supplement: Additional file 1 — Additional figures. Figure S1. Respiratory distress (RD) as a function of host age and rosetting. Figure S2. HB composition of known rosetting var genes. Figure S3. Linkage disequilibrium coefficient (D) values for all pairs of HBs in the genomic dataset. Figure S4. Community partition of weighted linkage network of HBs. Figure S5. HB-HB expression rate correlation matrix. Figure S6. Model of respiratory distress. Figure S7. Relationship between rosetting and respiratory distress. Figure S8. Relationship between impaired consciousness and the expression of various var types and HBs. Figure S9. The best fit relationship between six variables and rosetting using a window analysis. Figure S10. Relationship between rosetting and expression rates of var types and HBs. Figure S11. PC-classic var type association network. Figure S12. PC-HB relationships. Figure S13. Principal components in data space. Figure S14. The amount of variation explained by each PC. Figure S15. PCA for two subsets of the data. Figure S16. Representation of select homology blocks. Figure S17. HB-classic var type association network. [file 1471-2180-13-244-S1.pdf]

Supplementary Figures

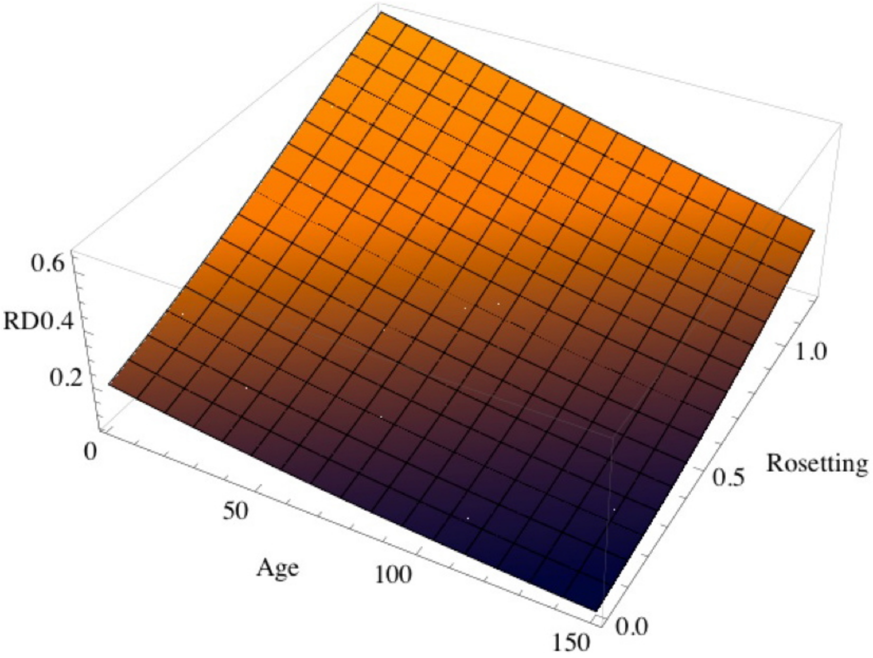

Figure S1. Respiratory distress (RD) as a function of host age and rosetting. The best fit logistic regression model of RD as a function of host age and rosetting is plotted. While there is a relationship between host age and several disease phenotypes, for this dataset we find that the influence of age on respiratory distress, impaired consciousness and rosetting is insignificant once models predicting these phenotypes incorporate sufficiently explanatory genetic variables.

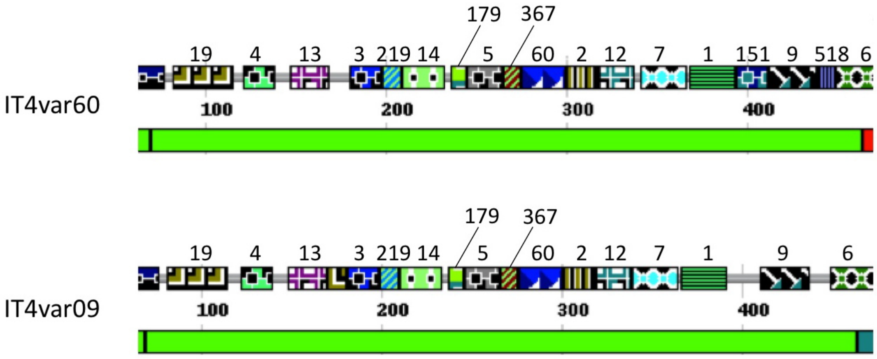

Figure S2. HB composition of known rosetting var genes. The HB composition of the DBLα domain of known rosetting *var* genes (D. Angeletti et al. 2012. Plasmodium falciparum rosetting epitopes converge in the SD3-loop of PfEMP1-DBL1alpha. PLoS One 7:e50758). Figures adapted from the output of VarDom web server.

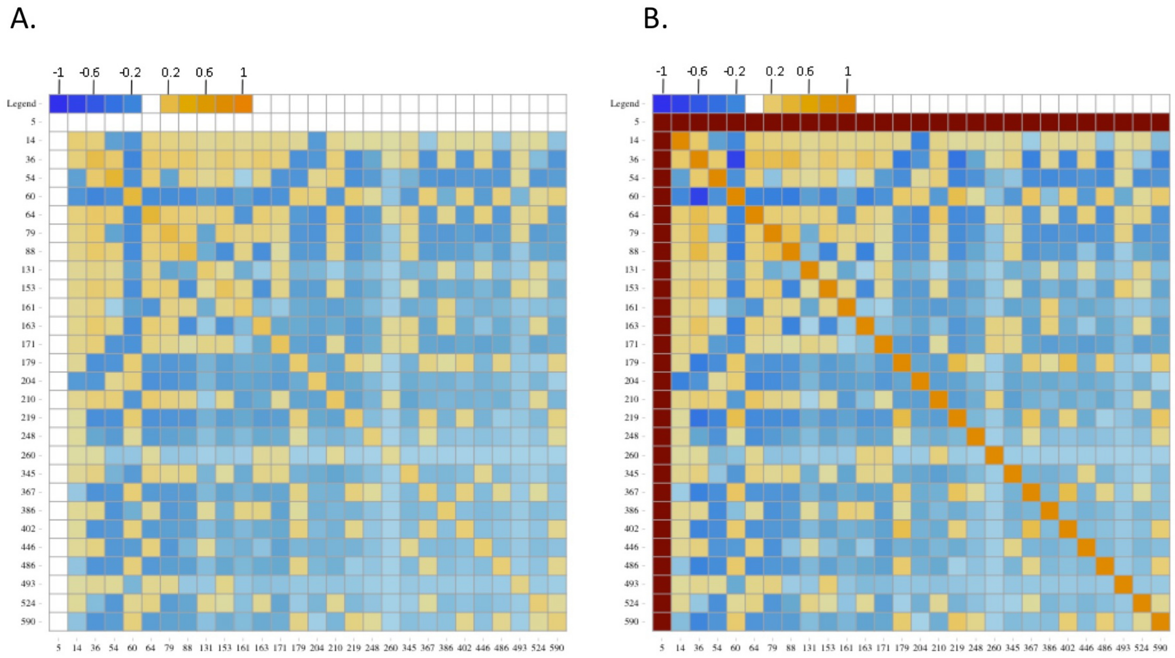

Figure S3. Linkage disequilibrium coefficient (D) values for all pairs of HBs in the genomic dataset. (A) Un-normalized D values. **(B)** D values normalized to account for the fact that D is maximized intermediate frequency alleles, with normalization being carried out by dividing D by (pq(1-p)(1-q))2, where p and q are the frequencies of the two HBs being compared. Dark red indicates indeterminate values for normalized D.

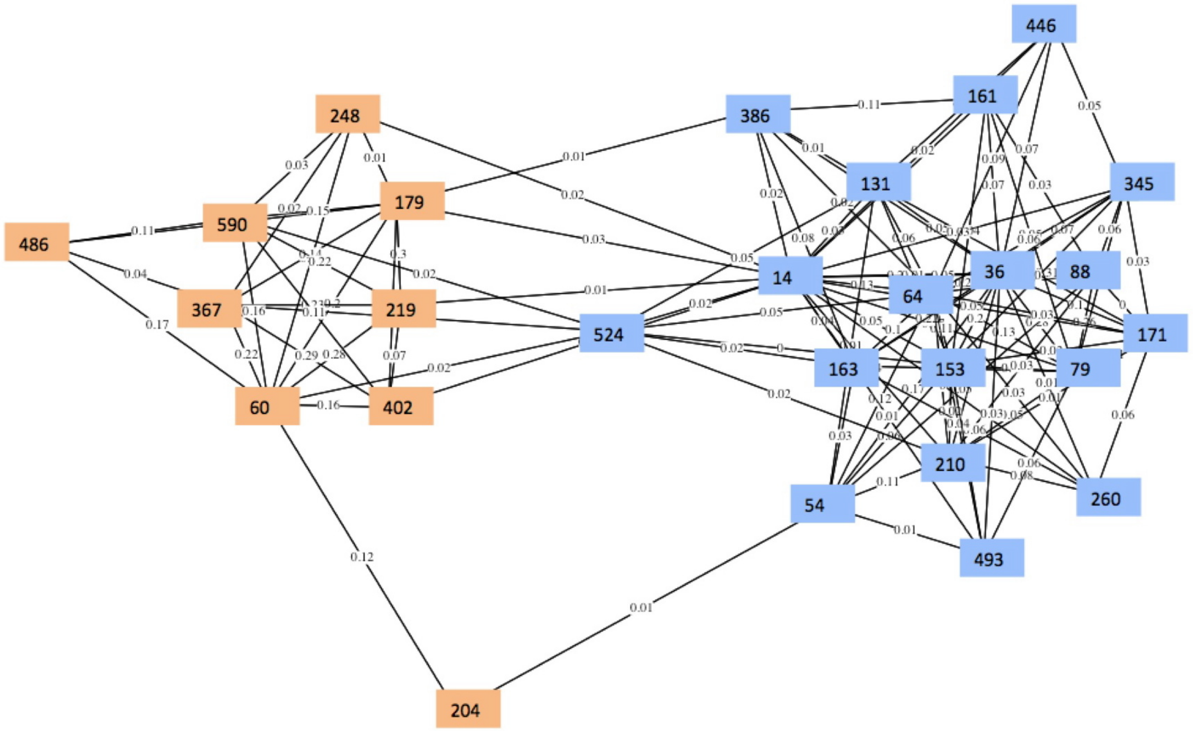

Figure S4. Community partition of weighted linkage network of HBs. Linkage weights are the positive linkage disequilibrium coefficient (D) values, normalized by HB frequency to account for the fact that D is maximized intermediate frequency alleles. Normalization is done here by dividing D by (pq(1-p)(1-q))2, where p and q are the frequencies of the two HBs. Community partition is carried out using the CommunityStructureAssignment function with consideration of edge weights. This is an unsupervised clustering algorithm– i.e., the number of groups is not predefined. Group assignment is reflected by node color. The third group, which is not shown, consists only of HB 5. The partition is carried out using the precise edge weights, however the rounded weights are shown.

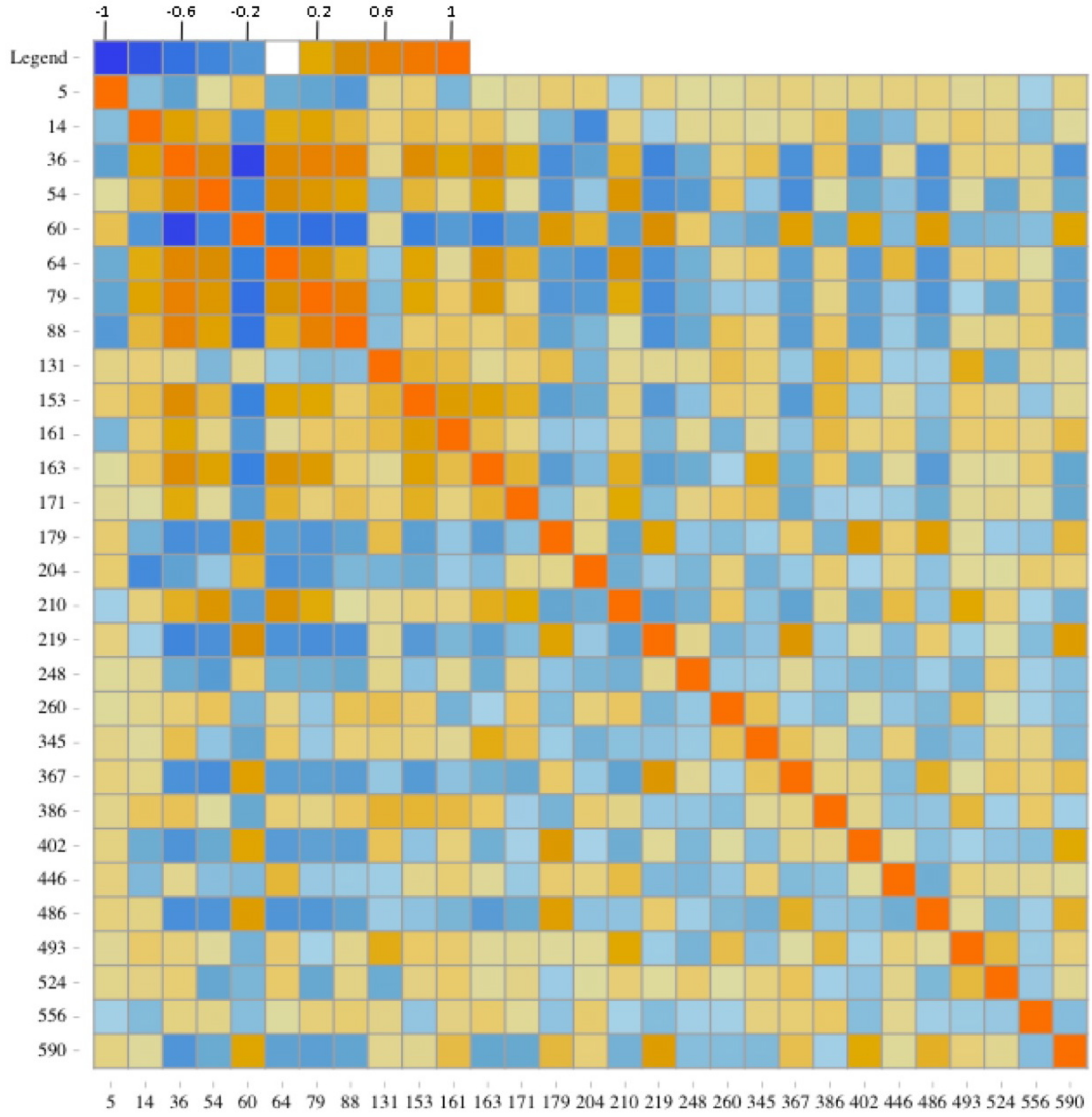

Figure S5. HB-HB expression rate correlation matrix. Shown are the Pearson's correlation coefficients between the expression rates of different HBs.

A.

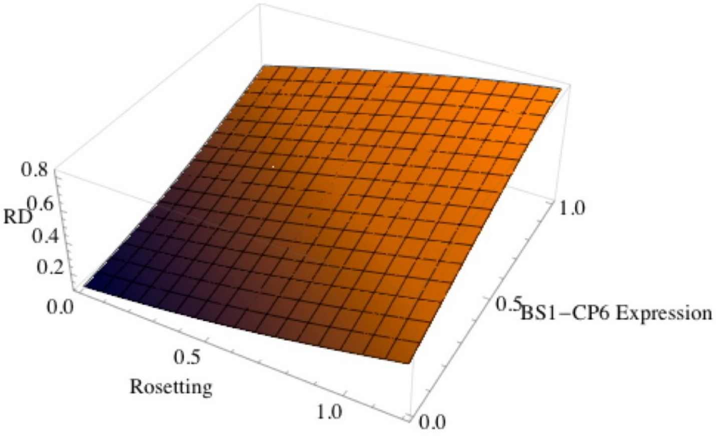

B.

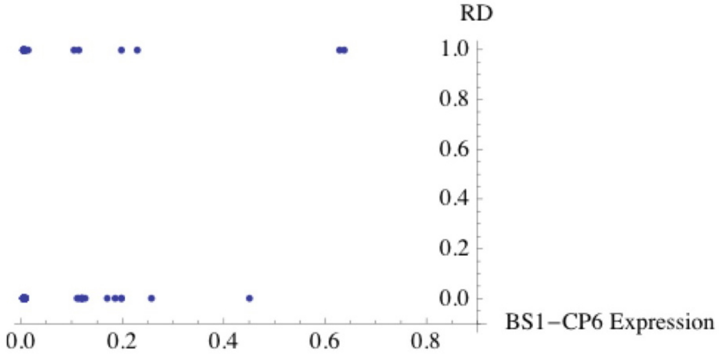

Figure S6. Model of respiratory distress. (A) A multiple logistic regression model of respiratory distress (RD) as a function of rosetting and BS1/CP6 expression rate. **(B)** The relationship between RD and BS1/CP6 expression rate.

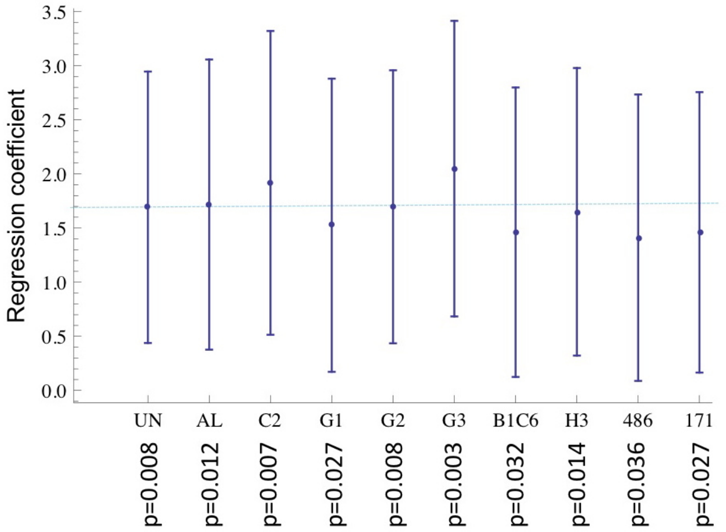

Figures S7. Relationship between rosetting and respiratory distress. The correlation coefficient for rosetting in a logistic regression model of respiratory distress after including age and the expression rate for a particular *var* type or HB in each model. Error bars represent 95% confidence intervals. UN=no added genetic variable, AL=group A-like, C2=cys2, G1=cysPoLV group 1, G2=cysPoLV group 2, G3=cysPoLV group 3, B1C6=BS1/CP6, H3=h3sub. The numbers refer to the expression rates for those particular HBs. Dotted line follows the estimate for the coefficient in the case of no added genetic variable.

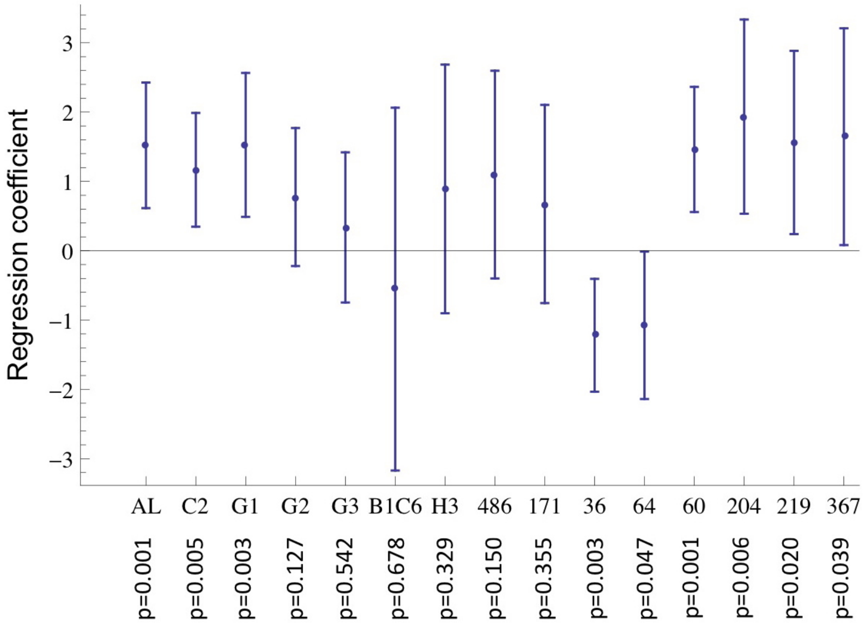

Figure S8. Relationship between impaired consciousness and the expression of various *var* types and HBs. The correlation coefficient for the expression rates for particular *var* types of HBs in a logistic regression model predicting impaired consciousness. All models also include host age as an independent variable. Error bars represent 95% confidence intervals. AL=group A-like, C2=cys2, G1=cysPoLV group 1, G2=cysPoLV group 2, G3=cysPoLV group 3, B1C6=BS1/CP6, H3=h3sub. The numbers refer to the expression rates for those particular HBs.

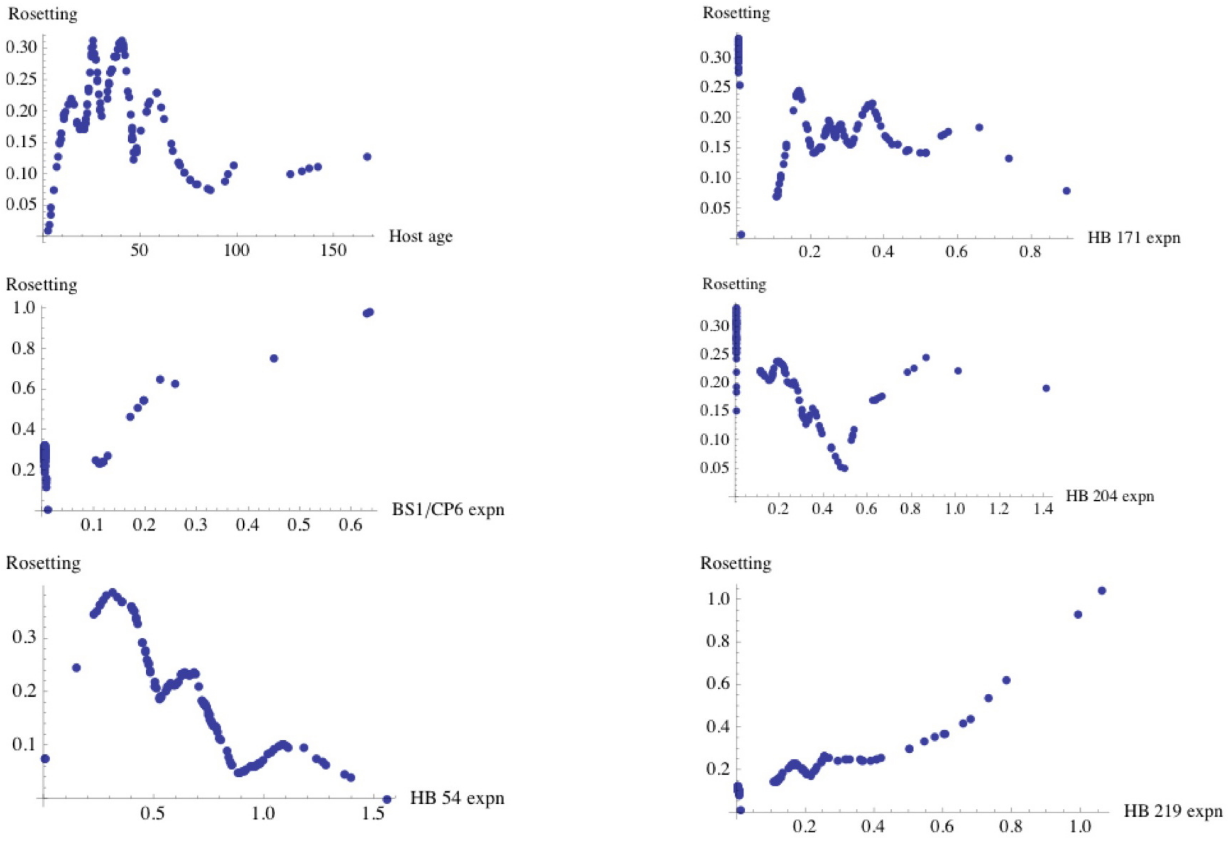

Figure S9. The best fit relationship between six variables and rosetting using a sliding window analysis. Shown are the six independent variables within a single multiple regression model of rosetting that includes host age. Sliding window analysis was carried out with the Lowess function in R with f=0.2.

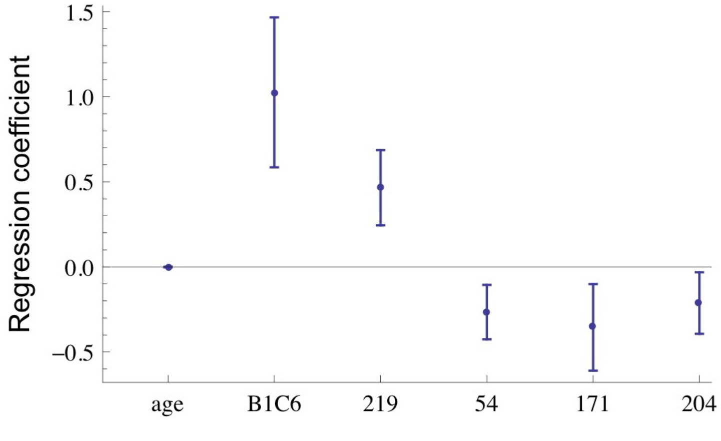

Figure S10. Relationships between rosetting and the expression rates of *var* types and HBs. The regression coefficients for the genetic variables that are part of a single multiple linear regression model of rosetting that includes host age. Error bars represent 95% confidence intervals. B1C6=BS1/CP6 *var* gene expression. The numbers refer to the expression rates for those particular HBs. This shows that the influence of age is not significant. Removing age from the model lowers the BIC score (to 34.07 from 37.34), reflecting an improved model.

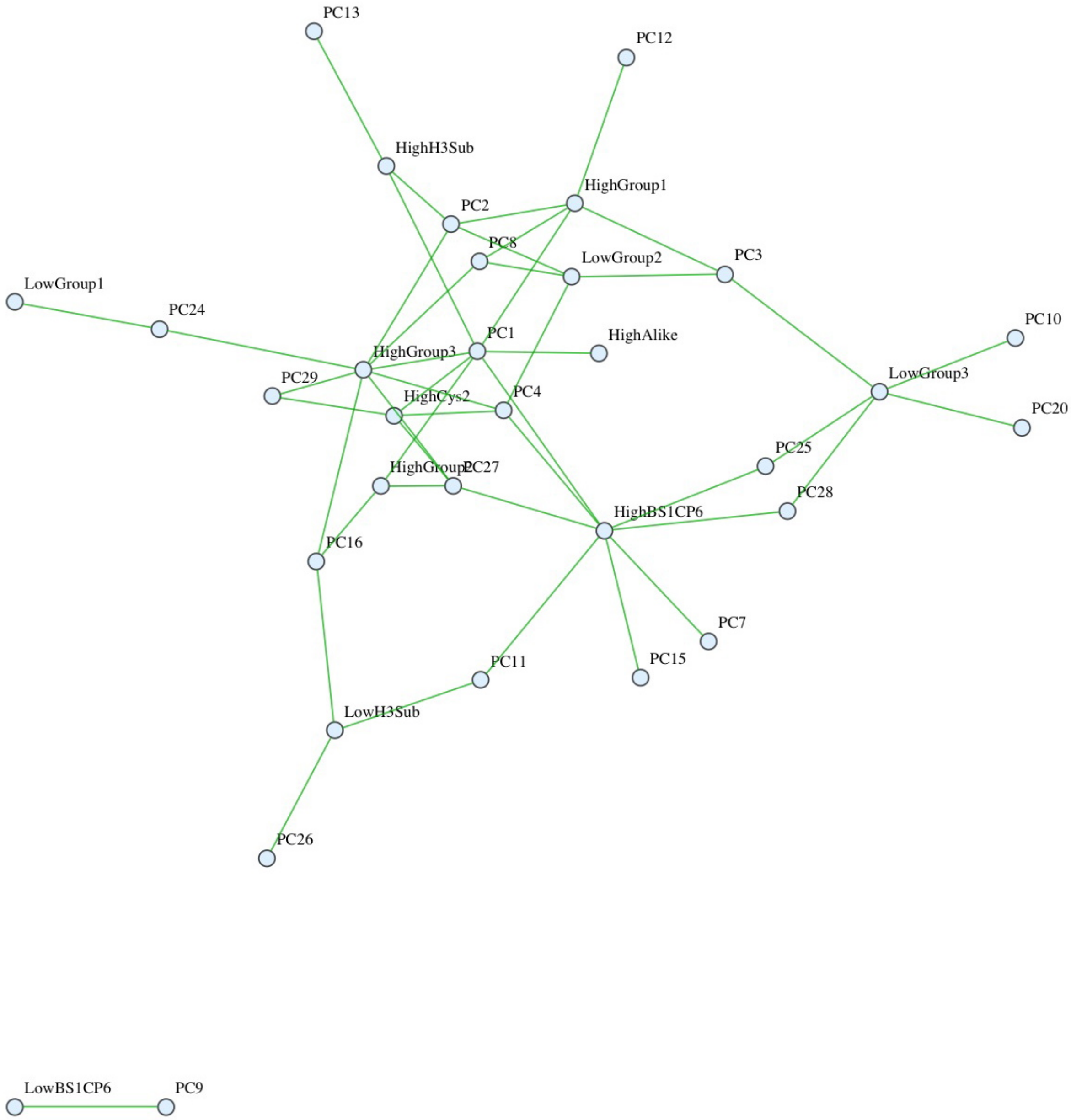

Figure S11. PC-classic *var* type association network. Network of significant correlations between HB expression rate profile principal components (PCs) and expression rates for classic *var* sequence types ($p \leq 0.05$).

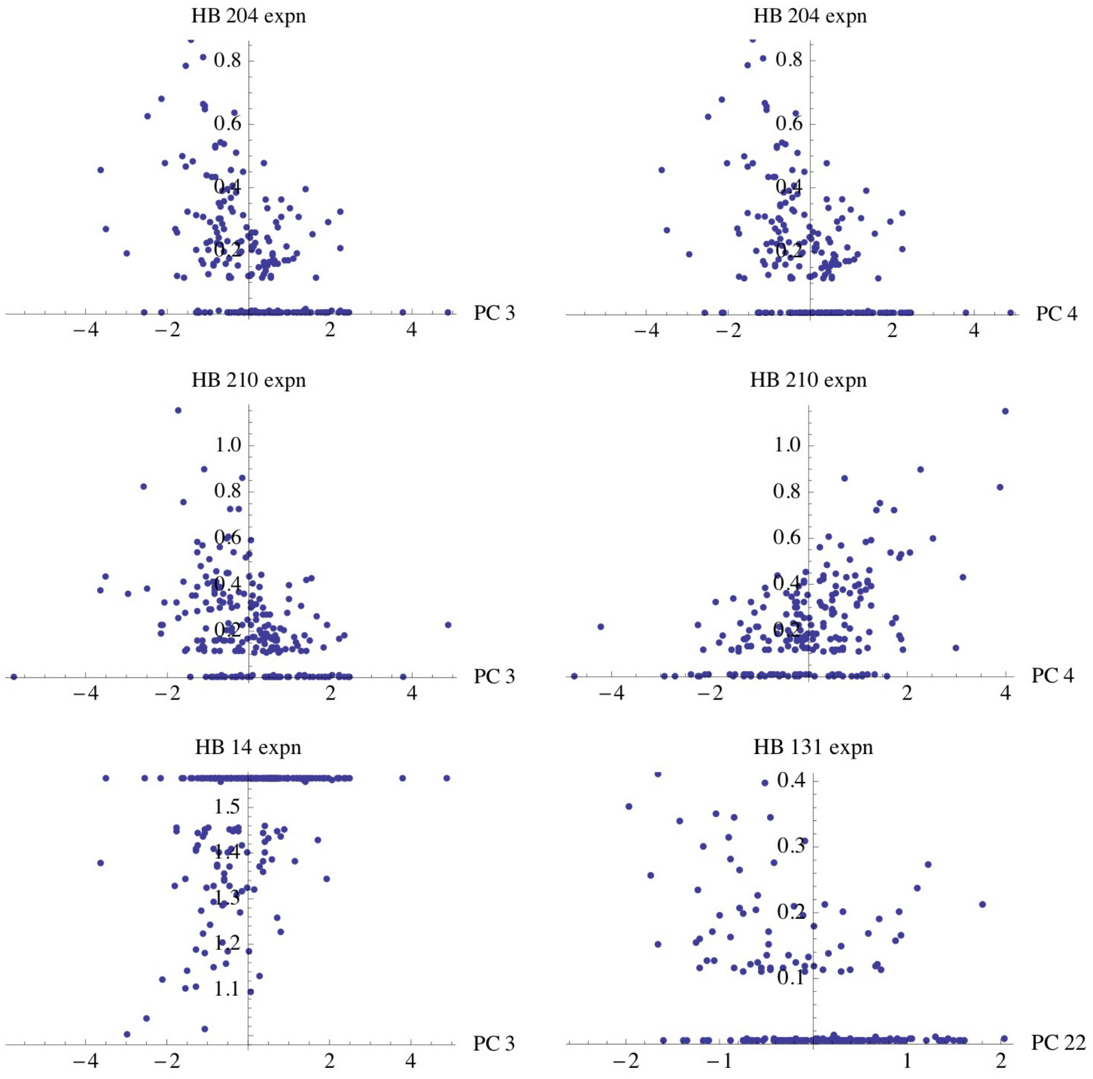

Figure S12. PC-HB relationships. The relationship between the expression rates of few particular HBs and a few of the principal components (PCs) of the HB expression rate profiles.

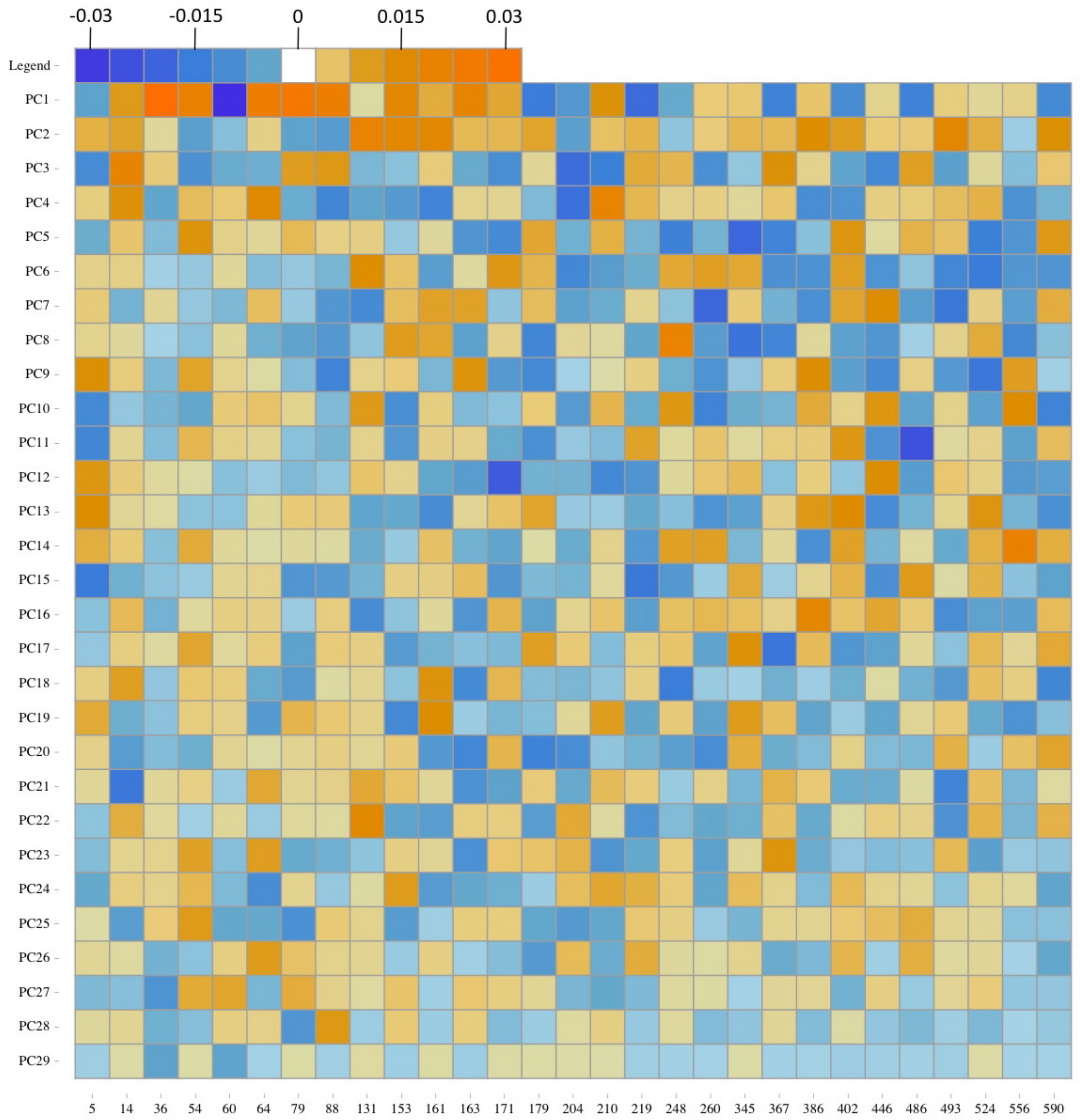

Figure S13. Principal components in data space. Principal components of HB expression rate profiles (PCs) in original data space of the individual HB expression rates, weighted by the singular values.

A.

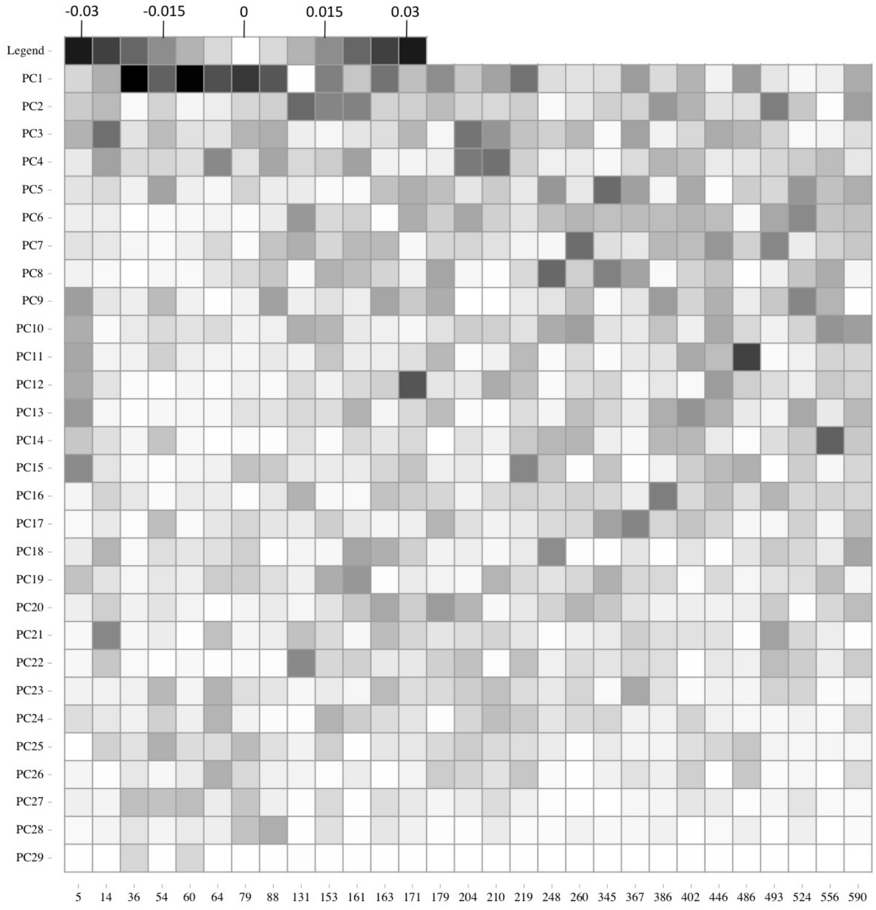

B.

PC1	0.0815
PC2	0.0482
PC3	0.0457
PC4	0.0439
PC5	0.0431
PC6	0.0407
PC7	0.0405
PC8	0.0401
PC9	0.0397
PC10	0.038
PC11	0.0373
PC12	0.0365
PC13	0.0355
PC14	0.0349
PC15	0.0343
PC16	0.0326
PC17	0.0318
PC18	0.0314
PC19	0.0314
PC20	0.0309
PC21	0.03
PC22	0.0287
PC23	0.0276
PC24	0.0256
PC25	0.0246
PC26	0.0214
PC27	0.0203
PC28	0.0164
PC29	0.0076

Figure S14. The amount of variation explained by each PC. (A) HB expression rate profile principal components (PCs) in the original data space of HB expression rates, weighted by the singular values. The absolute value of each coordinate is indicated in a grey-scale so that the amount of variation explained by each PC is visually evident. **(B)** The fraction of variation explained by each PC.

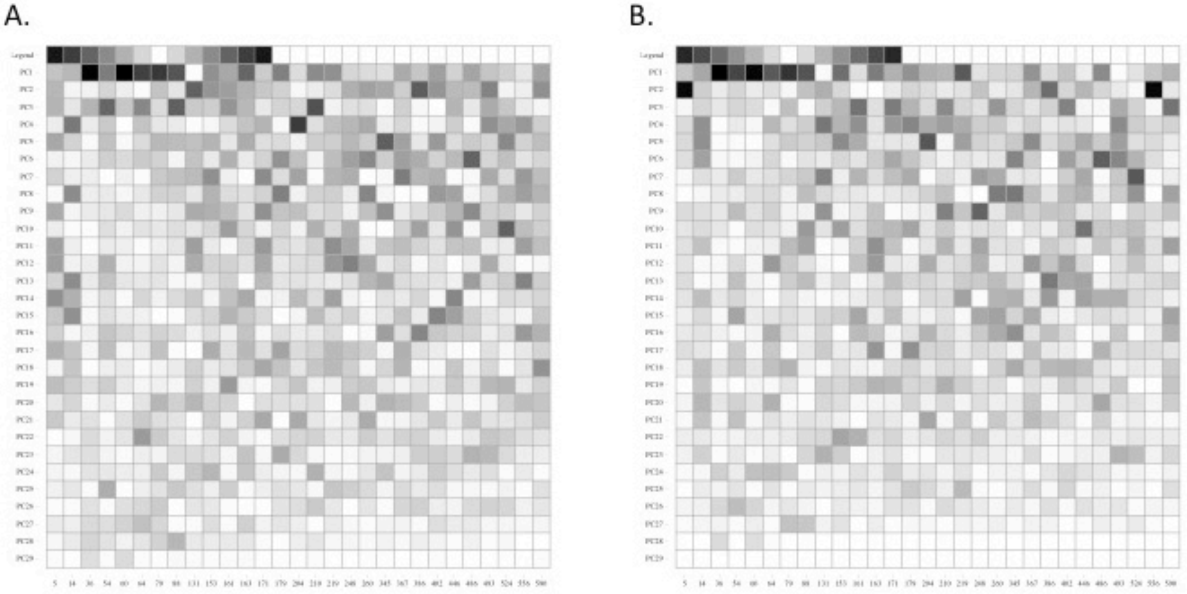

Figure S15. PCA for two subsets of the data. HB expression rate profile principal components (PCs) in the original data space of HB expression rates, weighted by the singular values. The absolute value of each coordinate is indicated in a grey-scale. In **(A)** a random subset of approximately half (n=108) of the 217 symptomatic isolates are used for the analysis, and in **(B)** the other approximate half (n=109) of the dataset is used for the PCA. PC 1 remains largely unaffected by the specific isolates used to carry out the PCA, since in the case of both partial datasets as well as the complete dataset, PC 1 is dominated by the expression rates of HB 36 and HB 60. PC 3 from the PCA of the complete dataset—which is dominated by the expression rate of HB 204—is observed in slightly different order with respect to other PCs when only half of the dataset is used at a time. In the case of **(A)**, the HB 204-dominated PC is PC 4, and in the case of **(B)**, the HB 204-dominated PC is PC 5.

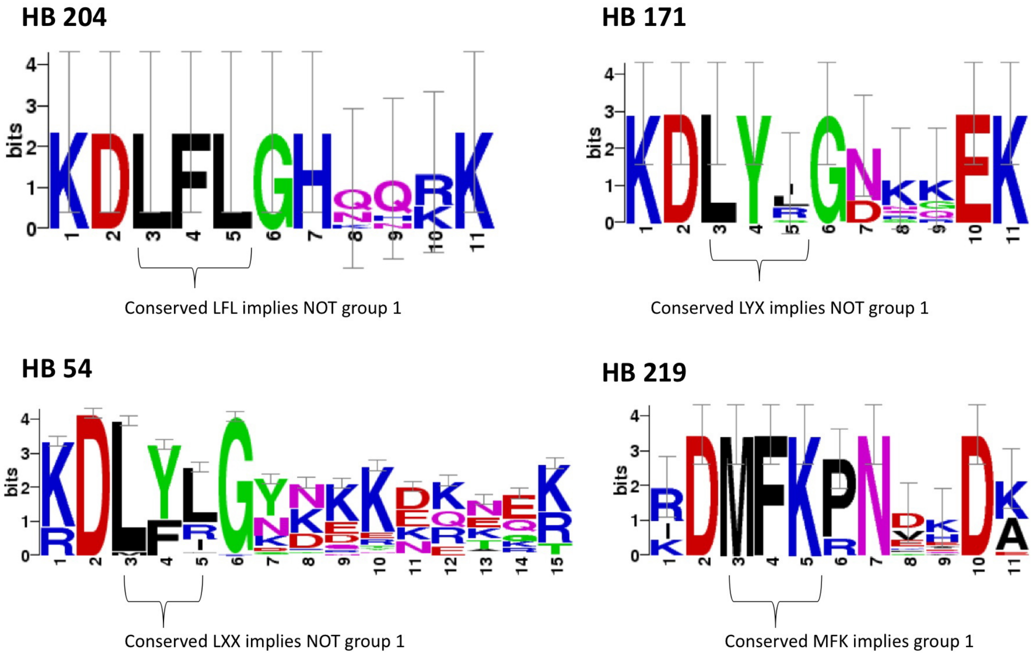

Figure S16. Representations of select homology blocks. Obtained from VarDom web server output with added annotations drawing connections to how they relate to some classic *var* tag types.

A.

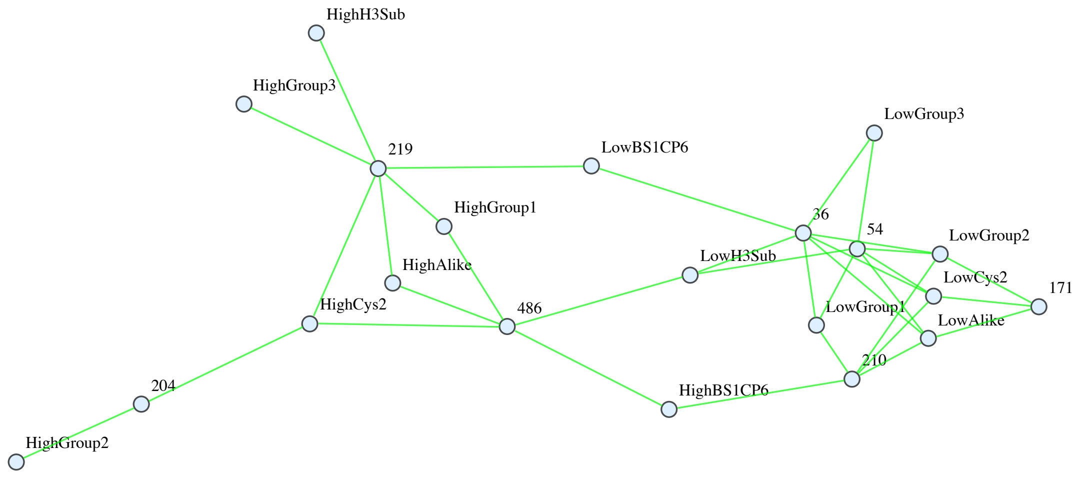

B.

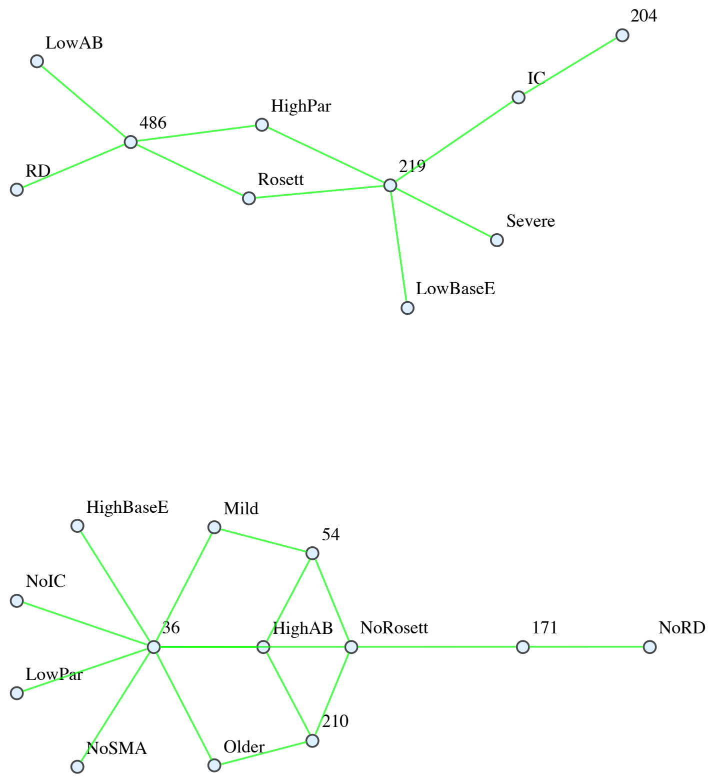

Figure S17. HB-classic *var* type association network. (A) The network of significant correlations between select HB expression rates and expression rates for classic *var* sequence types ($p \leq 0.05$). The HB expression rates included in the network are the ones that serve as dependent variables in Table I. **(B)** For the same set of select HBs, the network of significant correlations to disease phenotypes ($p \leq 0.05$).
