# Supplementary material for: Homology blocks of Plasmodium falciparum var genes and clinically distinct forms of severe malaria in a local population
Source: BMC Microbiol. 2013 Nov 6;13:244. doi: 10.1186/1471-2180-13-244 (PMC3827005; doi:10.1186/1471-2180-13-244)
Supplement: Additional file 2 — Further explanation of methods. [file 1471-2180-13-244-S2.pdf]

Further explanation of methods:

In an attempt to identify this hypothesized subset of *var* genes, Warimwe *et al*. (2012, Sci Transl Med 4:129ra45) used multiple regression models to test whether the expression rate of any previously defined subset of *var* tags could reduce the explanatory power of rosetting on RD. Their strongest candidate was a subset of tags that are simultaneously members of block-sharing group 1 (a group that corresponds to A-like *var* genes) and cysPoLV group 6. This class of genes will be referred to here as BS1/CP6. While the addition of the expression rate of this subset of *var* genes slightly reduced the explanatory power of rosetting on RD, its effect was small and statistically insignificant (Figure S6 in Additional File 1). Following a similar methodology, we tested whether HB expression rates could significantly reduce the explanatory power of rosetting on RD. We found that HB 171 expression had a similar effect to BS1/CP6 on the regression coefficient of rosetting (reducing it from 1.69 to 1.46), and that HB 486 had a larger effect (reducing it down to 1.41). However, even the expression rate of HB 486 did not reduce the coefficient for rosetting below the lower boundary of its original confidence interval, at 0.438, so neither constitutes a significant result (Figure S7 in Additional File 1). As in the case of BS1/CP6, neither HB 486 nor HB 171 expression is correlated with IC in this dataset (Figure S8 in Additional File 1). Although no *var* type expression rate or HB expression rate significantly reduces the explanatory power of rosetting as a predictor of RD, it is still possible that there is a genetic marker among these *var* tags that is associated with this particular type of severe disease. In fact, the above test has relatively low power to detect the hypothesized group of *var* genes (see Methods), and that is why we turn to a different methodology.
