# Supplementary material for: Homology blocks of Plasmodium falciparum var genes and clinically distinct forms of severe malaria in a local population
Source: BMC Microbiol. 2013 Nov 6;13:244. doi: 10.1186/1471-2180-13-244 (PMC3827005; doi:10.1186/1471-2180-13-244)
Supplement: Additional file 3 — Additional tables. Table S1. Multiple regression models of rosetting that include an HB expression rate as an independent variable. Table S2. Multiple regression models of rosetting that include an HB expression PC as an independent variable. Table S3. Statistics for multiple regression models predicting rosetting with and without age. [file 1471-2180-13-244-S3.pdf]

Supplementary tables

Table SI. Multiple regression models of rosetting that include an HB expression rate as an independent variable*

Independent Variables	AIC	BIC	R^2	Adjusted R^2
Age,AL,C2,G1,G2,G3,B1C6,H3	21.9684	50.7204	0.387784	0.347639
Age,AL,C2,G1,G2,G3,B1C6,H3,5	23.9512	55.5784	0.387865	0.342334
Age,AL,C2,G1,G2,G3,B1C6,H3,14	22.843	54.4702	0.393021	0.347874
Age,AL,C2,G1,G2,G3,B1C6,H3,36	23.4921	55.1192	0.390006	0.344635
Age,AL,C2,G1,G2,G3,B1C6,H3,54	19.0223	50.6495	0.410469	0.366619
Age,AL,C2,G1,G2,G3,B1C6,H3,60	23.4298	55.057	0.390296	0.344946
Age,AL,C2,G1,G2,G3,B1C6,H3,64	23.5873	55.2145	0.389563	0.344158
Age,AL,C2,G1,G2,G3,B1C6,H3,79	23.7366	55.3638	0.388867	0.34341
Age,AL,C2,G1,G2,G3,B1C6,H3,88	23.7337	55.3608	0.38888	0.343425
Age,AL,C2,G1,G2,G3,B1C6,H3,131	23.4345	55.0617	0.390274	0.344923
Age,AL,C2,G1,G2,G3,B1C6,H3,153	23.4217	55.0488	0.390334	0.344987
Age,AL,C2,G1,G2,G3,B1C6,H3,161	23.859	55.4862	0.388295	0.342797
Age,AL,C2,G1,G2,G3,B1C6,H3,163	23.8943	55.5215	0.388131	0.34262
Age,AL,C2,G1,G2,G3,B1C6,H3,171	22.0522	53.6793	0.396675	0.351799
Age,AL,C2,G1,G2,G3,B1C6,H3,179	22.6246	54.2517	0.394033	0.348961
Age,AL,C2,G1,G2,G3,B1C6,H3,204	16.9953	48.6225	0.41952	0.376344
Age,AL,C2,G1,G2,G3,B1C6,H3,210	21.9282	53.5553	0.397245	0.352412
Age,AL,C2,G1,G2,G3,B1C6,H3,219	22.5738	54.201	0.394267	0.349213
Age,AL,C2,G1,G2,G3,B1C6,H3,248	23.928	55.5552	0.387973	0.34245
Age,AL,C2,G1,G2,G3,B1C6,H3,260	23.699	55.3262	0.389042	0.343599
Age,AL,C2,G1,G2,G3,B1C6,H3,345	22.3893	54.0164	0.39512	0.350129
Age,AL,C2,G1,G2,G3,B1C6,H3,367	23.5754	55.2026	0.389618	0.344218
Age,AL,C2,G1,G2,G3,B1C6,H3,386	23.4898	55.117	0.390017	0.344646
Age,AL,C2,G1,G2,G3,B1C6,H3,402	23.8821	55.5092	0.388188	0.342681
Age,AL,C2,G1,G2,G3,B1C6,H3,446	23.1223	54.7495	0.391726	0.346482
Age,AL,C2,G1,G2,G3,B1C6,H3,486	23.5557	55.1828	0.38971	0.344317
Age,AL,C2,G1,G2,G3,B1C6,H3,493	22.8114	54.4386	0.393168	0.348031
Age,AL,C2,G1,G2,G3,B1C6,H3,524	23.7102	55.3374	0.38899	0.343543
Age,AL,C2,G1,G2,G3,B1C6,H3,556	23.2254	54.8525	0.391247	0.345968
Age,AL,C2,G1,G2,G3,B1C6,H3,590	23.2902	54.9174	0.390946	0.345644

*Highlighted cases are those where the model improves with the addition of an HB expression rate.
AL=group A-like, C2=cys-2, G1=cysPoLV group 1, G2=cysPoLV group 2, G3=cysPoLV group 3, B1C6=BS1/CP6, H3=h3sub.

Table SII. Multiple regression models of rosetting that include an HB expression PC as an independent variable*

Independent Variables	AIC	BIC	R^2	Adjusted R^2
Age,AL,C2,G1,G2,G3,B1C6,H3	21.9684	50.7204	0.387784	0.347639
Age,AL,C2,G1,G2,G3,B1C6,H3,PC1	22.4941	54.1213	0.394636	0.349609
Age,AL,C2,G1,G2,G3,B1C6,H3,PC2	23.921	55.5481	0.388006	0.342486
Age,AL,C2,G1,G2,G3,B1C6,H3,PC3	16.7279	48.3551	0.420704	0.377616
Age,AL,C2,G1,G2,G3,B1C6,H3,PC4	23.1699	54.7971	0.391505	0.346245
Age,AL,C2,G1,G2,G3,B1C6,H3,PC5	23.2716	54.8988	0.391032	0.345737
Age,AL,C2,G1,G2,G3,B1C6,H3,PC6	23.9621	55.5893	0.387814	0.342279
Age,AL,C2,G1,G2,G3,B1C6,H3,PC7	23.9475	55.5747	0.387882	0.342353
Age,AL,C2,G1,G2,G3,B1C6,H3,PC8	23.939	55.5661	0.387922	0.342396
Age,AL,C2,G1,G2,G3,B1C6,H3,PC9	23.9396	55.5668	0.387919	0.342392
Age,AL,C2,G1,G2,G3,B1C6,H3,PC10	23.9464	55.5736	0.387887	0.342358
Age,AL,C2,G1,G2,G3,B1C6,H3,PC11	23.8382	55.4653	0.388393	0.342901
Age,AL,C2,G1,G2,G3,B1C6,H3,PC12	21.325	52.9522	0.400014	0.355387
Age,AL,C2,G1,G2,G3,B1C6,H3,PC13	23.3138	54.941	0.390836	0.345526
Age,AL,C2,G1,G2,G3,B1C6,H3,PC14	22.3988	54.026	0.395076	0.350082
Age,AL,C2,G1,G2,G3,B1C6,H3,PC15	23.2751	54.9022	0.391016	0.34572
Age,AL,C2,G1,G2,G3,B1C6,H3,PC16	23.8542	55.4813	0.388318	0.342821
Age,AL,C2,G1,G2,G3,B1C6,H3,PC17	23.5312	55.1584	0.389824	0.344439
Age,AL,C2,G1,G2,G3,B1C6,H3,PC18	23.9418	55.569	0.387909	0.342381
Age,AL,C2,G1,G2,G3,B1C6,H3,PC19	23.8015	55.4287	0.388564	0.343085
Age,AL,C2,G1,G2,G3,B1C6,H3,PC20	21.0748	52.7019	0.401159	0.356617
Age,AL,C2,G1,G2,G3,B1C6,H3,PC21	23.1689	54.796	0.39151	0.34625
Age,AL,C2,G1,G2,G3,B1C6,H3,PC22	20.7867	52.4139	0.402475	0.358031
Age,AL,C2,G1,G2,G3,B1C6,H3,PC23	22.5104	54.1375	0.394561	0.349528
Age,AL,C2,G1,G2,G3,B1C6,H3,PC24	23.1642	54.7914	0.391531	0.346273
Age,AL,C2,G1,G2,G3,B1C6,H3,PC25	23.9579	55.585	0.387834	0.342301
Age,AL,C2,G1,G2,G3,B1C6,H3,PC26	23.5258	55.153	0.389849	0.344466
Age,AL,C2,G1,G2,G3,B1C6,H3,PC27	23.8281	55.4553	0.38844	0.342952
Age,AL,C2,G1,G2,G3,B1C6,H3,PC28	21.7344	53.3616	0.398136	0.353369
Age,AL,C2,G1,G2,G3,B1C6,H3,PC29	22.9089	54.536	0.392716	0.347546

*Highlighted case is one where the model improves with the addition of a PC.
AL=group A-like, C2=cys-2, G1=cysPoLV group 1, G2=cysPoLV group 2, G3=cysPoLV group 3, B1C6=BS1/CP6, H3=h3sub

Table SIII. Statistics for multiple regression models predicting rosetting with and without age*

	Independent variables	AIC	BIC	R^2	Adj. R^2
A	Age,Cys2,Grp1,Grp2,Grp3,BS1CP6	20.97	41.09	0.364	0.339
	Cys2,Grp1,Grp2,Grp3,BS1CP6	20.14	37.40	0.358	0.338
B	Age, HB36, HB204, HB210, HB219, HB486	18.46	41.46	0.385	0.356
	HB36, HB204, HB210, HB219, HB486	16.48	36.60	0.385	0.361
C	Age, BS1CP6, HB54, HB171, HB204, HB219	14.34	37.34	0.405	0.376
	BS1CP6, HB54, HB171, HB204, HB219	16.82	34.07	0.374	0.354
D	Age, BS1CP6, PC1, PC3, PC4, PC22	6.505	29.51	0.439	0.412
	BS1CP6, PC1, PC3, PC4, PC22	4.776	24.90	0.438	0.415

*The result of removing the least significant genetic variable, one by one, from models of rosetting that start with the expression rates of: (row A) the 7 classic *var* types, (row B) the 29 HB expression rates, (row C) the expression rates for both the 7 classic *var* types and the 29 HBs, and (row D) the expression rates for the 7 classic *var* types and the 29 HB expression rate PCs. The variable selection procedure is done maintaining host age in the model, however statistics are shown for the optimal model with age (green rows) and after removing age (white rows).
